# Comprehensive ubiquitome analysis reveals persistent mitochondrial remodeling disruptions from doxorubicin-induced cardiotoxicity in aged CD-1 male mice

**DOI:** 10.1007/s00204-025-04006-2

**Published:** 2025-03-04

**Authors:** Sofia Reis Brandão, Elisa Lazzari, Rui Vitorino, Germana Meroni, Ana Reis-Mendes, Maria João Neuparth, Francisco Amado, Félix Carvalho, Rita Ferreira, Vera Marisa Costa

**Affiliations:** 1 Associate Laboratory i4HB - Institute for Health and Bioeconomy, Faculty of Pharmacy, University of Porto, 4050-313 Porto, Portugal; 2 UCIBIO-Applied Molecular Biosciences Unit, Laboratory of Toxicology, Department of Biological Sciences, Faculty of Pharmacy, University of Porto, 4050-313 Porto, Portugal; 3 LAQV-REQUIMTE, Department of Chemistry, University of Aveiro, 3810-193 Aveiro, Portugal; 4 Molecular Genetics Lab, Department of Life Sciences, University of Trieste, 34127 Trieste, Italy; 5 Institute of Biomedicine (Ibimed), Department of Medical Sciences, University of Aveiro, 3810-193 Aveiro, Portugal; 6 Department of Surgery and Physiology, Faculty of Medicine, UnIC@RISE, University of Porto, 4200-319 Porto, Portugal; 7 Laboratory for Integrative and Translational Research in Population Health (ITR), Research Centre in Physical Activity, Health and Leisure (CIAFEL), Faculty of Sports, University of Porto, 4200-450 Porto, Portugal; 8 UCIBIO - Applied Molecular Biosciences Unit, Translational Toxicology Research Laboratory, University Institute of Health Sciences (1H-TOXRUN, IUCS-CESPU), 4585-116 Gandra, Portugal; 9 Present Address: LAQV-REQUIMTE, Laboratory of Bromatology and Hydrology, Department of Chemical Sciences, Faculty of Pharmacy, University of Porto, 4050-313, Porto, Portugal

**Keywords:** Old, Anticancer therapy, Cardio-oncology, Chronic cardiotoxicity, Ubiquitination, Mitochondrial dynamics

## Abstract

Doxorubicin (DOX)-associated cardiotoxicity is characterized by long-term manifestations, whose mechanisms remain incompletely understood, and is exacerbated by various risk factors, with age being a prominent contributor. The objective of this study was to assess the enduring cardiac molecular impacts of DOX in old CD-1 male mice, focusing on ubiquitinated proteins. At 19 months of age, DOX group received a cumulative dose of 9.0 mg/kg of DOX, while control animals got saline solution. Animals were sacrificed 2 months after the administration. DOX induced heart structural changes and increased proteolytic activity. Additionally, increased protein ubiquitination was observed in DOX group, despite the decreased content of the E3 ubiquitin-protein ligase Atrogin-1. A search of poly-ubiquitinated proteins, enriched by tandem ubiquitin-binding entities (TUBEs), showed increased poly-ubiquitination of proteins associated with sarcomere organization and mitochondrial metabolism processes by DOX. Increased mitochondrial density inferred by higher citrate synthase activity was found in DOX group. Moreover, decreased biogenesis and auto(mito)phagy occurred in DOX animals, proven by decreased peroxisome proliferator-activated receptor γ coactivator 1 α, Beclin1 and microtubule-associated protein light chain 3 content. These findings indicate a reduction in mitochondrial biogenesis and accumulation of dysfunctional mitochondria in the aged heart, along with elevated levels of poly-ubiquitinated proteins after DOX treatment. Thus, the disruption of mitochondrial remodeling and impaired protein ubiquitination emerge as enduring consequences of DOX-induced cardiotoxicity, persisting for even 2 months after DOX exposure. This underscores the long-lasting impact of DOX, with significant effects continuing beyond the period of administration, which advocates for longer clinical surveillance.

**Supplementary Information:**

The online version contains supplementary material available at 10.1007/s00204-025-04006-2.

## Introduction

Doxorubicin (DOX) stands out as one of the most widely used anticancer drugs to treat solid tumors and hematologic malignancies, such as leukemias and lymphomas. This chemotherapeutic agent primarily exerts its action by inhibiting topoisomerase II (TOP2), interrupting replication and transcription processes (Löwenberg et al. 1998; Citron et al. 2003; Seiter 2005). While DOX has proven to have a remarkable success in anticancer therapy, severe adverse effects have been reported, such as myelosuppression and cardiotoxicity (Seiter 2005). Indeed, cardiotoxicity related to chemotherapeutic agents is the second cause of death among cancer patients, having an important impact on their quality of life and life span (Seiter 2005; Ewer and Ewer 2010; Strongman et al. 2022).

The cardiotoxicity of DOX may manifest at the onset of treatment (up to two weeks after its conclusion) as arrhythmias, acute coronary syndromes, acute heart failure, or pericarditis/myocarditis. In this scenario, it is categorized as acute/subacute (Seiter 2005; Colombo and Cardinale 2013). When the clinical cardiac manifestations occur within 1 year (early-onset) or more years (late-onset) after treatment, they are categorized as chronic cardiotoxicity, featuring decreased left ventricular ejection fraction, and eventually congestive heart failure. This chronic cardiotoxicity typically entails irreversible modifications (Colombo and Cardinale 2013; Jain and Aronow 2019). Most studies have focused on acute/subacute cardiotoxic models, even after single doses (Merten et al. 2005; Cui et al. 2010; Xi et al. 2011; Brandão et al. 2022), which may not consistently align with the extensive clinical use of DOX. Indeed, the chronic form of DOX cardiotoxicity is the most predominant among survivors, being dose related. A lifelong cumulative maximum recommended dosage ranging from 400 to 550 mg/m^2^ has been established for DOX, to limit the cardiotoxic effects and to improve the quality of life of post-cancer survivors (Reis-Mendes et al. 2015; Viñas-Mendieta et al. 2024). On the other hand, the success of anticancer therapy, to which DOX made a large contribution, has led to huge increases in survivorship rates (Siegel et al. 2019; Viñas-Mendieta et al. 2024), whose patients need to face the consequences of the aggressive therapies being used. In this context, there is presently a critical need to assess the cardiac changes occurring longer after the exposure, to better understand the late-onset chronic cardiotoxicity.

Cancer is a disease that predominantly affects the elderly population, and the number of cancer survivors is increasing as stated above. Even so, the clinical studies usually do not investigate this population regarding neither survival nor the adverse effects, namely cardiotoxicity (Anker et al. 2024). However, current perspectives suggest that the age at diagnosis and preexistent comorbidities have been associated with increased DOX cardiotoxicity (Von Hoff 1979; Reis-Mendes et al. 2015). Usually, older people are already more prone to suffer from comorbidities related to cardiovascular diseases, including obesity and diabetes, as well as pre-existing cardiac conditions, so it is assumed that they have fewer cardiac reserves (Grann et al. 2006; Zamorano et al. 2016; Anker et al. 2024). For this reason, 70 years old and older patients are regularly not included in clinical trials and, when included, only the healthiest ones received chemotherapeutic agents and in lower cumulative doses than the younger patients (Swain et al. 2003; Grann et al. 2006; Todaro et al. 2013). Thus, the cancer treatments used in elderly patients are less aggressive than the ones used in adult patients. Consequently, a less effectivity of DOX may occur in older patients, leading to higher mortality by cancer (Swain et al. 2003; Grann et al. 2006). Moreover, the follow-up of cancer patients is often confined to monitoring cancer recurrence, leading to a lack of awareness regarding early signs of cardiotoxicity and allowing irreversible alterations to happen, whose mechanisms are not yet completely understood. Even in non-clinical models, the cardiotoxicity studies are poorly represented in old animals (Reis-Mendes et al. 2023).

Thus, there is an unmet need to understand if the old heart acts similarly to a putative DOX insult when taking a clinically relevant experimental paradigm. The first mechanisms proposed for DOX-induced cardiotoxicity were related to increased production of reactive oxygen species (ROS), and the concomitant lipid peroxidation, protein modification, DNA and mitochondrial damage, along with disturbance of calcium homeostasis (Davies and Doroshow 1986; Ichikawa et al. 2014; Reis-Mendes et al. 2015; Brandão et al. 2022). Nowadays, the idea that the accumulation of damaged proteins, firstly considered as a consequence of oxidative stress, has been challenged. Indeed, the activation of proteolytic systems, including ubiquitin–proteasome pathway (UPP), calpain system, and autophagy, along with other signaling pathways, dependent or independent of cardiac TOP2 β inhibition, have been reported as putative cardiotoxic mechanisms of DOX (Renu et al. 2018; Abrahams et al. 2022; Brandão et al. 2022; Gaytan et al. 2023). In addition, cardiac aging is influenced by analogous pathways, including autophagy and proteolytic systems (Sun et al. 2016; Boengler et al. 2017; Ghosh et al. 2020).

Therefore, the main objective of the present study was to assess the impact of protein ubiquitination and the UPP on the long-term effects induced by DOX in the aged heart. We also aimed to search for other proteolytic systems implicated in DOX-induced cardiac remodeling. To address this, elderly CD-1 male mice, approximately 19 months old, were employed as a non-clinical model, being that these mice received a clinically relevant cumulative dose of DOX (9 mg/kg), given in multiple doses as occurs in clinical settings.

## Materials and methods

### Animal experimental paradigm

Male CD-1 mice (*Mus musculus*), 42–48 days old, were obtained from Charles River Laboratories (L’Arbresle, France) and kept in the Institute of Biomedical Sciences Abel Salazar (ICBAS-UP) rodent animal house facility. The welfare of the animals was monitored daily by expert veterinarians, following the conditions and procedures previously described (Brandão et al. 2021). The animal protocol was approved by the local Committee Responsible for Animal Welfare (ORBEA) of ICBAS-UP (project nº 140/2015) and national competent authorities (General Directorate of Food and Veterinary, DGAV, reference nº 0421/000/000/2016), and followed the European Council Directive (2010/63/EU).

The total cumulative dose of 9 mg/kg of DOX hydrochloride (Sigma-Aldrich, St. Louis, MO, USA) was clinically relevant as seen by allometric scaling and using the body area surface conversion factor between mice and humans, 37, as recommended by the US Food and Drug Administration (Reagan‐Shaw et al. 2008). This DOX dosage corresponds roughly to ~ 55 mg/m^2^ in humans, which is inferior to the 400–550 mg/m^2^ maximum lifelong dose recommended for humans (Reis-Mendes et al. 2015).

The protocol started when the mice were 19 months old, approximately equivalent to 75 years old in humans (Wang et al. 2020). Mice were separated into two groups, control (CTRL) and DOX, and received six intraperitoneal (i.p.) injections, distributed biweekly for 3 weeks. The DOX animals received six i.p. injections of 1.5 mg/kg of DOX, prepared in saline solution (0.9% NaCl) and in sterile conditions. The CTRL animals received six i.p. injections of 0.9% NaCl in the same volume and conditions as the DOX group. This administration program was implemented to mimic human anticancer therapy, in which multiple administrations at separated time points are given to the patients (Dores-Sousa et al. 2015). Two months after the last i.p. injection, mice were anesthetized through isoflurane inhalation and sacrificed by exsanguination, and the morphometric parameters, such as whole-body weight, heart weight and tibial length, were assessed. At sacrifice, the animals exhibited an age (~ 22 months old) equivalent of approximately 79 human years old (Wang et al. 2020).

### Blood collection and serum analysis

During euthanasia, the abdominal cavity was opened to expose the inferior vena cava, and the blood sample was collected in commercial tubes containing an inert clotting agent (Vetlab ZT Plain + Gel for Serum 1.1 ml Fill). The blood was allowed to clot at room temperature for 45 min. Afterwards, the clot was pelleted by centrifugation (900 g, 10 min, room temperature), and the obtained serum was aliquoted and stored at − 80 °C. An aliquot was analyzed for total protein, albumin, glucose, and cholesterol concentration, as well as creatine kinase-MB (CK-MB) activity, employing an AutoAnalyzer (Prestige 24i, Cormay PZ, Diamond Diagnostics, Holliston, MA, USA).

### Heart collection and histological analysis

After blood collection, the chest cavity was opened, and the hearts were rapidly excised, carefully dried, and weighed. Heart apex was divided for histological assessment, as recently reported (Brandão et al. 2023). Briefly, after fixation with paraformaldehyde, the apex was dehydrated with grade ethanol solutions, cleared with xylene, and finally embedded in paraffin. Then, the slides obtained were stained with hematoxylin and eosin to assess tissue damage, as previously described (Dores-Sousa et al. 2015). A Carl Zeiss Imager A1 light microscope equipped with an AxioCam MRc 5 digital camera (Oberkochen, Baden-Württemberg, Germany) was used for morphology assessment.

### Heart homogenization

The heart ventricular part was homogenized in lysis buffer [0.5 mM ethylene glycol-bis(2-aminoethylether)-N, N, N′, N′-tetraacetic acid (EGTA, Sigma-Aldrich), 10 mM HEPES hemisodium salt (Sigma-Aldrich), pH 7.4, 0.1% (v/v) Triton X-100 (Sigma-Aldrich), with protease inhibitors cocktail (1:400, P8340, Sigma-Aldrich) and phenylmethanesulfonyl fluoride (PMSF, 1:1000, Sigma-Aldrich); 50 mg of tissue/mL of buffer] using a Teflon pestle on a tight-fitting Potter–Elvehjem glass homogenizer at 0–4 °C. Cardiac homogenates were aliquoted. One of the samples was immediately used for the measurement of protein content, applying the DC Protein Assay (Bio-Rad, Hercules, CA, USA) method with bovine serum albumin (BSA) as standard, and following manufacturer’s guidelines. The remaining samples were stored at − 80 °C until further analysis.

### Western blot analysis

Cardiac content of the E3 ubiquitin-protein ligase Atrogin-1 (also known as muscle atrophy F-box), autophagy protein 5 (ATG5), B-cell lymphoma-2 interacting protein 3 (BNIP3), Beclin1, Cbp/p300-interacting transactivator 4 (CITED4), CCAAT/enhancer-binding protein β (C/EBPβ), mast/stem cell growth factor receptor Kit (SCFR), microtubule-associated protein light chain 3 (LC3B), mitochondrial transcription factor A (Tfam), mitofusin 1 (Mfn1), muscle RING finger protein 1 (MuRF1), Parkin, and peroxisome proliferator-activated receptor γ coactivator 1 α (PGC-1α) was assessed by Western blot. Protein ubiquitination and carbonylation levels were also estimated by Western blot, using ubiquitin and dinitrophenyl hydrazone product specific antibodies (for more details consult Supplementary Table S1).

Homogenized cardiac protein (30 μg, or 50 μg for MuRF1 and protein ubiquitination levels) were run on 12.5% SDS-PAGE following Laemmli work (Laemmli 1970). For protein carbonylation levels assessment, a given volume (V, corresponding to 20 μg of protein) was mixed with 1 V of 12% sodium dodecyl sulfate (SDS), 2 V of 2 mM 2,4-dinitrophenylhydrazine (DNPH)/10% trifluoroacetic acid (TFA), and incubated in dark for 30 min until the addition of 1.5 V of reducing buffer (2 M Tris-base, 18% of β-mercaptoethanol, 30% of glycerol) to stop the reaction. The derivatized proteins were loaded without boiling on a 12.5% SDS-PAGE following Laemmli (Laemmli 1970).

After gel separation, proteins were blotted onto a nitrocellulose membrane (Amersham Protran, GE Healthcare, Germany) for 2 h (protein carbonylation and ubiquitination levels) or 1 h (searched proteins) at 200 mA in transfer buffer (5 mM Tris, 192 mM glycine, pH 8.5, 20% [v/v] methanol). Ponceau S staining (except for protein carbonylation levels) was used for controlling the loading, and the obtained images are indicated in Supplementary Figure S1.

Membranes were blocked with 5% (w/v) nonfat dry milk in Tris-buffered saline with Tween 20 (TBS-T) for 1 h. Next, membranes followed an incubation overnight at 4 °C with the primary antibodies diluted 1:500 or 1:1000 in 5% (w/v) nonfat dry milk in TBS-T (consult Supplementary Table S1 for detailed info of the antibodies and specific dilutions used). Washing of membranes was performed with TBS-T (3 × 10 min). Afterwards, membranes were incubated with the appropriate secondary horseradish peroxidase-conjugated antibody (anti-mouse [NA931] or anti-rabbit [NA934], GE Healthcare, Buckinghamshire, UK) diluted 1:1000 in 5% (w/v) nonfat dry milk in TBS-T for 2 h at room temperature. After washing (3 × 10 min of TBS-T), membranes were exposed to enhanced chemiluminescence (ECL) reagent (Bio-Rad) following the manufacturer’s recommendations. The immunoreactive bands were detected using the ChemiDoc Imaging System (Bio-Rad) and the images obtained were analyzed with Image Lab software version 6.0.1 (Bio-Rad). Results are presented in arbitrary units of optical density with a representative blot for each group. The complete blots obtained are indicated in Supplementary Figure S2.

### Determination of proteolytic activity by zymography

Protein (50 μg) from cardiac homogenates was mixed with non-reducing sample buffer (0.1 M Tris–HCl pH 6.8, 5% [w/v] SDS, 20% [v/v] glycerol and 0.1% [w/v] bromophenol blue) and then, loaded without boiling onto a 10% SDS-PAGE filled with 0.1% (w/v) porcine gelatin. After electrophoresis, gels were incubated in a renaturation buffer (2.5% [v/v] Triton X-100) for 90 min at room temperature with soft agitation. Then, gels were incubated in a developing buffer (50 mM Tris–HCl pH 7.4, 5 mM NaCl, 10 mM CaCl_2_, 10 mM ZnCl_2_, 0.25% [v/v] Triton X-100) for 30 min. Afterwards, gels were incubated in fresh developing buffer overnight at 37 °C as previously described (Caseiro et al. 2012). In parallel, gels were incubated in a developing buffer containing matrix metalloproteinases (MMPs) inhibitors (50 mM Tris–HCl pH 7.4, 5 mM NaCl, 10 mM ethylenediaminetetraacetic acid (EDTA), 0.25% [v/v] Triton X-100) for 30 min and then overnight at 37 °C. After that, all gels were stained with 0.5% (w/v) Coomassie Brilliant Blue G250, and then washed in destaining solution (25% [v/v] ethanol, 5% [v/v] acetic acid) until evident contrast between bands resulting from proteolytic activity and the gel background was observed. The gels were scanned using the ChemiDoc Imaging System (Bio-Rad) and analyzed with Image Lab software version 6.0.1 (Bio-Rad). Results are presented in arbitrary units of optical density with a representative lane for each group. The complete gels scanned are presented in Supplementary Figure S3.

### Pull-down of poly-ubiquitinated proteins through tandem ubiquitin-binding entities (TUBEs)

Poly-ubiquitinated protein pools were obtained using cross-linked glutathione S-transferase (GST)-TUBEs as this method optimizes the capture of ubiquitinated proteins (Aillet et al. 2012). Therefore, recombinant GST-tagged TUBEs and GST (to be used as negative control), both already covalently linked to glutathione resin, were purchased from Life Sensor Inc. (Malvern, PA, USA). The following procedures were performed according to the protocol described in Xolalpa et al. (2016). Cardiac homogenates (900 μg) were diluted in TUBEs lysis buffer (TLB; 20 mM Na_2_HPO_4_, 20 mM NaH_2_PO_4_, 1% IGEPAL, 2 mM EDTA, 500 mM NaF, 5 mM Na pyrophosphate tetrabasic, 10 mM β-glycerol phosphate disodium salt pentahydrate, pH 7.5) supplemented with inhibitors of deubiquitylating enzymes (PR619, 50 μM) and proteasome (MG132, 20 μM). Cardiac lysates were incubated with 30 μL cross-linked GST resin for 45 min at 4 °C with agitation (pre-clearing step) and the unbound fraction was incubated with 30 μL cross-linked GST-TUBEs resin for a further 45 min at 4 °C with agitation (capture step). TUBEs unbound fraction was removed and both pre-clearing and capture resins were washed three times with TLB, three times with phosphate-buffered saline containing 0.05% (v/v) Tween 20, and twice with TLB containing 1 M NaCl. Resin-bound proteins were eluted in Laemmli buffer and analyzed by immunoblot alongside input and unbound fractions for the presence of ubiquitinated proteins. The blots obtained are presented in Supplementary Figure S4 and indicate the successful enrichment of poly-ubiquitinated proteins in GST-TUBEs fraction. All fractions were stored at −80 °C until further analyses.

### SDS-PAGE followed by liquid chromatography coupled to tandem mass spectrometry (GeLC-MS/MS) analysis of pulled-down poly-ubiquitinated proteins

Equal volumes of samples containing the enriched poly-ubiquitinated proteins (GST-TUBEs fraction; six animals per group) were separated using a 12.5% SDS-PAGE as previously described by Laemmli (Laemmli 1970). Gels were incubated with fixing solution (40% [v/v] methanol, 10% [v/v] glacial acetic acid) for 35 min at room temperature, rinsed with deionized water quickly, and incubated with 0.5% (w/v) Coomassie Brilliant Blue G250 overnight at room temperature to reach good staining. Then, gels were rinsed with deionized water quickly and destained with 25% (v/v) methanol until an optimal contrast was achieved. The gels were scanned using the ChemiDoc Imaging System (Bio-Rad) and are presented in Supplementary Figure S5. Next, protein bands were cut off from the gels and *in-gel* digested with trypsin to be analyzed by LC–MS/MS, as previously described (Shevchenko et al. 2006; Brandão et al. 2021). In brief, gel pieces were washed with 25 mM ammonium bicarbonate and acetonitrile. Then, gel pieces were reduced using 10 mM 1,4-dithiothreitol and alkylated with 55 mM iodoacetamide. Finally, the gel pieces were incubated overnight at 37 °C with modified porcine trypsin (1:20 [w/w] enzyme-to-total protein ratio) prepared in 50 mM ammonium bicarbonate.

The tryptic peptides were extracted using 5% (v/v) formic acid (FA), and subsequently with a solution of 5% (v/v) FA with 50% (v/v) acetonitrile. The resulting peptide mixture was dried using a SpeedVac (Savant, SPD121P, Thermo Fischer Scientific, Northumberland, UK) and resuspended in 1% (v/v) FA. The dried peptides were analyzed with a QExactive Orbitrap (Thermo Fisher Scientific, Bremen, Germany) through the EASY-spray nano ESI source (Thermo Fisher Scientific) that was coupled to an Ultimate 3000 (Dionex, Sunnyvale, CA) high-pressure liquid chromatography (HPLC) system. The trap column (100 μm I.D. × 2 cm packed with Acclaim PepMap RSLC C18, 5 μm 100 Å) and the EASY-spray analytical column (75 μm I.D. × 75 cm packed with Acclaim PepMap RSLC C18, 3 μm 100 Å) were from Thermo Fisher Scientific. Peptides were trapped in 96% solvent A (0.1% [v/v] FA) at 30 μL/min. The elution was completed using solvent B (0.1% [v/v] FA with 80% [v/v] acetonitrile) at 300 nL/min. The gradient was completed as follows: from 0 to 3 min utilizing 96% solvent A, from 3 to 70 min utilizing 4–25% solvent B, from 70 to 90 min utilizing 25–40% solvent B, from 90 to 92 min utilizing 90% solvent B, from 92 to 100 min utilizing 90% solvent B, and from 101 to 120 min utilizing 96% solvent A. Then, the MS analysis was performed in data-dependent acquisition mode at 1.8 kV. The MS/MS method was applied with an FT survey scan from 400 to 1600 m/z, a resolution of 70,000, and auto gain control (AGC) target 1E6. Only the 10 most intense peaks were exposed to higher-energy collisional dissociation with a resolution of 17,500, AGC target 5E4, normalized collision energy 28%, and 100 ms of max injection time with a dynamic exclusion of 35 s, to obtain MS/MS spectrum.

For identification and label-free quantification (LFQ) of peptides, the MaxQuant software (version 1.6.5.0) was employed. The MS/MS spectra were searched against the UniprotKB/Swiss-Prot protein sequence database under *Mus musculus* (July 2023 version) applying Andromeda. The variable modifications chosen were methionine oxidation (M), deamidation (NQ), and ubiquitination (GlyGly). Spectra manual inspection was performed to confirm the presence of the modified Lys amino acid residue (GlyGly) in some of the peptides and consequently check the attribution of protein ubiquitination modification. An example of a representative spectrum of a poly-ubiquitinated peptide from ATP synthase alpha chain is given in Supplementary Figure S6, highlighting the GlyGly modification. The mass tolerance for the precursor mass and fragments were 20 ppm and 0.15 Da, respectively. Five amino acids length was specified as the lowest peptide extent, and two as the highest failed cleavages. The false discovery rate (FDR) for identification was defined as 1%. Only proteins identified by at least two peptides were considered. The protein quantification was attributed based on the LFQ of the most intense peptide and present in at least four out of the six samples of each group. The mass spectrometry proteomics data have been deposited to the ProteomeXchange Consortium via the PRIDE (Perez-Riverol et al. 2022) partner repository with the dataset identifier PXD048078.

The identification of the biological processes for each protein was attributed according to the biological process using Gene Ontology database on Uniprot (http://www.uniprot.org). Jvenn (Bardou et al. 2014) was used to obtain the Venn diagram distribution of the proteins amongst the groups. Raw data were log-transformed before the principal component analysis (PCA) and partial least squares discriminant analysis (PLS-DA) that were used to examine proteome dynamics between groups. The missing values were replaced by 1/5 of the minimum positive values of their corresponding variables. Volcano plot was used to annotate proteins that were present with significantly different variations between the groups. These analyses were performed using the MetaboAnalyst v5.0 web portal (http://www.metaboanalyst.ca, accessed on 11 July 2023).

## Determination of citrate synthase (CS) activity

Cardiac CS activity was measured in cardiac homogenates following the established literature protocol (Coore et al. 1971). The 5,5′-dithiobis-(2-nitrobenzoic acid) reacts with the free thiol groups of coenzyme A (CoA), which results from the reaction of oxaloacetate with acetyl-CoA sodium salt. The resulting 2-nitro-5-thiolbenzoate anion was read at 412 nm (molar extinction coefficient of 13.6 mM^−1.^cm^−1^) during approximately 2 min at 30 °C in a microplate reader (Multiskan GO, Thermo Fischer Scientific, Northumberland, UK). The values were normalized to total protein.

### Statistical analysis

The data are presented as mean ± standard deviation (SD). The statistical analysis was performed with GraphPad Prism software (version 6.0.1, GraphPad Software Inc, La Jolla, CA, USA) and the experimental groups were compared using a two-sided *t*-test, unless otherwise mentioned in the figure legend. No multiple testing corrections were performed. Results were considered statistically significant when *p*-value < 0.05 and a trend was acknowledged for *p*-values between 0.05 and 0.1.

## Results

### Heart structural changes happened 2 months after the DOX administration

DOX treatment had no substantial impact on the body weight or heart mass (Supplementary Table S2). Still, DOX mice showed edema and ascites, which may have suppressed the differences in the evaluated parameters. Indeed, a deeper search demonstrated that 44% of mice in the DOX group (four out of the nine) revealed a decrease in whole-body weight higher than 5%, which is indicative of overall body wasting, often considered a consequence of cancer therapy (Tisdale 2009). Nonetheless, a high mortality (> 20%) was observed in the overall animals, as the advanced age of animals at the beginning of protocol (19 months old) and the considerable time elapsed after the administration period (2 months) made natural death an important factor during this experimental paradigm (Zamorano et al. 2016). The effect of DOX exposure on serum markers was also assessed (Supplementary Table S2). DOX treatment had no meaningful impact on the serum levels of total protein, albumin, glucose, and cholesterol or the activity of CK-MB, a marker of cardiac injury. Overall, no significant changes were observed in systemic parameters 2 months after the cessation of DOX exposure.

**Fig. 1:**
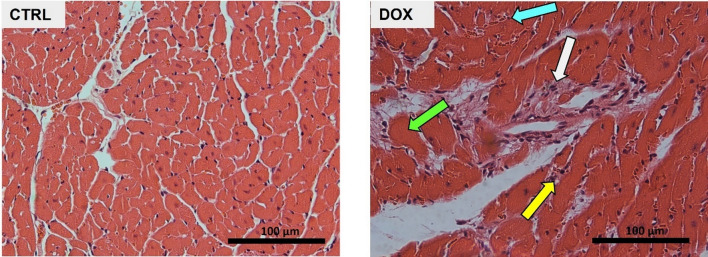
DOX impact on heart structure assessed through light microscopy. Representative light micrographs obtained by hematoxylin and eosin staining from CTRL and DOX mice are depicted (*n* = 3). The CTRL group showed normal morphology and structure. DOX group showed vascular congestion (cyan arrow), inflammatory infiltration (white arrow), necrotic zones (green arrow), and vacuolization (yellow arrow). Images were taken at 40 × magnification (scale bar = 100 µm)

In contrast, the histological analysis of the heart showed structural changes, even 2 months after DOX exposure (Fig. 1). CTRL mice presented typical morphology and structure of the myocardium with regular cell distribution, while DOX showed increased vacuolization of the cytoplasm and several necrotic zones, along with evident infiltration of inflammatory cells, vascular congestion, and edema at cellular and interstitial levels. Moreover, DOX treatment led to dispersed areas of connective tissue with abundant fibroblast proliferation and collagen deposition, which were semi-quantified in another study of our group (Reis-Mendes et al. 2023), suggesting interstitial cardiac fibrosis. These findings highlighted cardiac disorganization caused by DOX even 2 months after therapy cessation, which indicates heart maladaptive remodeling. Additionally, molecular markers associated with heart regeneration (SCFR, C/EBPβ and CITED4) were assessed in cardiac tissue but no significant differences were observed between the two groups (Supplementary Figure S7a). These results suggest no major impact of DOX on heart regeneration 2 months after the treatment, although heart structural alteration is still present at this stage (Fig. 1).

### Cardiac content of ubiquitinated proteins and proteolysis were impacted 2 months after the DOX administration

**Fig. 2:**
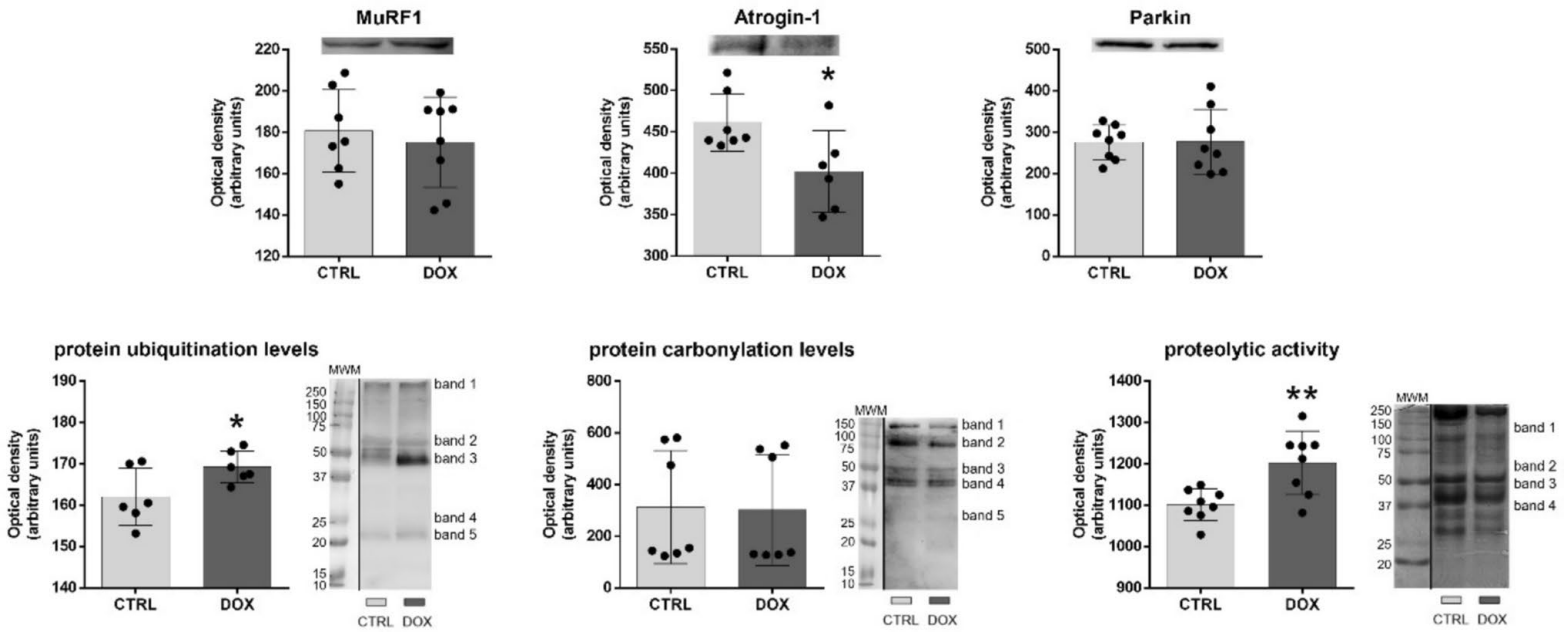
DOX impact on heart E3 ubiquitin-protein ligases (MuRF1, Atrogin-1, Parkin), protein ubiquitination and carbonylation levels, along with proteolytic activity. A representative image of the Western blots obtained is presented. The molecular weight, in kDa, of each band of the molecular weight marker (MWM) observed is depicted for protein ubiquitination and carbonylation levels, and proteolytic activity. For the total blots and proteolytic gels obtained see Supplementary Figure S2 and S3, respectively. For the optical density of each band of protein ubiquitination and carbonylation levels, as well as proteolytic activity see Supplementary Figure S7b. All datapoints are presented overlaying with the mean ± SD (*n* = 6–8) of content. Experimental groups were statistically compared using the unpaired two-sided *t*-test: *p < 0.05, **p < 0.01. *MuRF1* muscle RING finger protein 1

Given that DOX treatment has been linked to the accumulation of misfolded proteins in the heart (Renu et al. 2018; Brandão et al. 2022), which are not efficiently cleared by the UPP, one of the primary proteolytic systems in the heart (Montalvo et al. 2020; Gaytan et al. 2023), the E3 ubiquitin-protein ligases MuRF1, Atrogin-1 and Parkin were assessed in cardiac homogenates (Fig. 2). MuRF1 content was not changed in the DOX group, but the content of Atrogin-1 showed a significant decrease, suggesting a lower protein ubiquitination and clearance by this specific E3 ubiquitin-protein ligase (Peris-Moreno et al. 2020; Singh et al. 2021). Additionally, no differences were found for Parkin, which acts as an E3 ubiquitin-protein ligase mainly for mitochondrial targets (Singh et al. 2021).

Moreover, the overall content of ubiquitinated proteins was also evaluated, and increased levels were found in the DOX group (Fig. 2). Nonetheless, no differences were found in protein carbonylation levels, indicating that the accumulation of ubiquitinated proteins observed is not associated with the buildup of oxidatively modified proteins, 2 months after the DOX treatment.

In addition, the whole cardiac proteolytic activity was assessed through zymography, and increased activity was noticed in the DOX group (Fig. 2). The zymography gels, in which inhibitors of MMPs were included, showed bands of similar molecular weight and intensity to those in the gels without the inhibitors. This indicates that the observed proteolytic activity does not originate from MMPs, but rather from other protease families. Specifically, four bands were identified in the zymography gels, and based on their molecular weight and information from Uniprot, we can speculate that calpain-3 (94 kDa, band 1), cathepsin F (52 kDa, band 2), cathepsin D (45 kDa, band 3), and cathepsin B (37 kDa, band 4) may contribute to the observed increased proteolytic activity in cardiac muscle. In detail, the activity in these four bands was higher in DOX-treated mice even 2 months after the end of treatment (Supplementary Figure S7b). Thus, DOX treatment increased proteolytic activity and ubiquitination levels in proteins, indicating increased cardiac remodeling that aligns with the heart damage caused by this anthracycline (Fig. 1).

### Cardiac mitochondrial proteins are more susceptible to ubiquitination 2 months after DOX administration

**Fig. 3:**
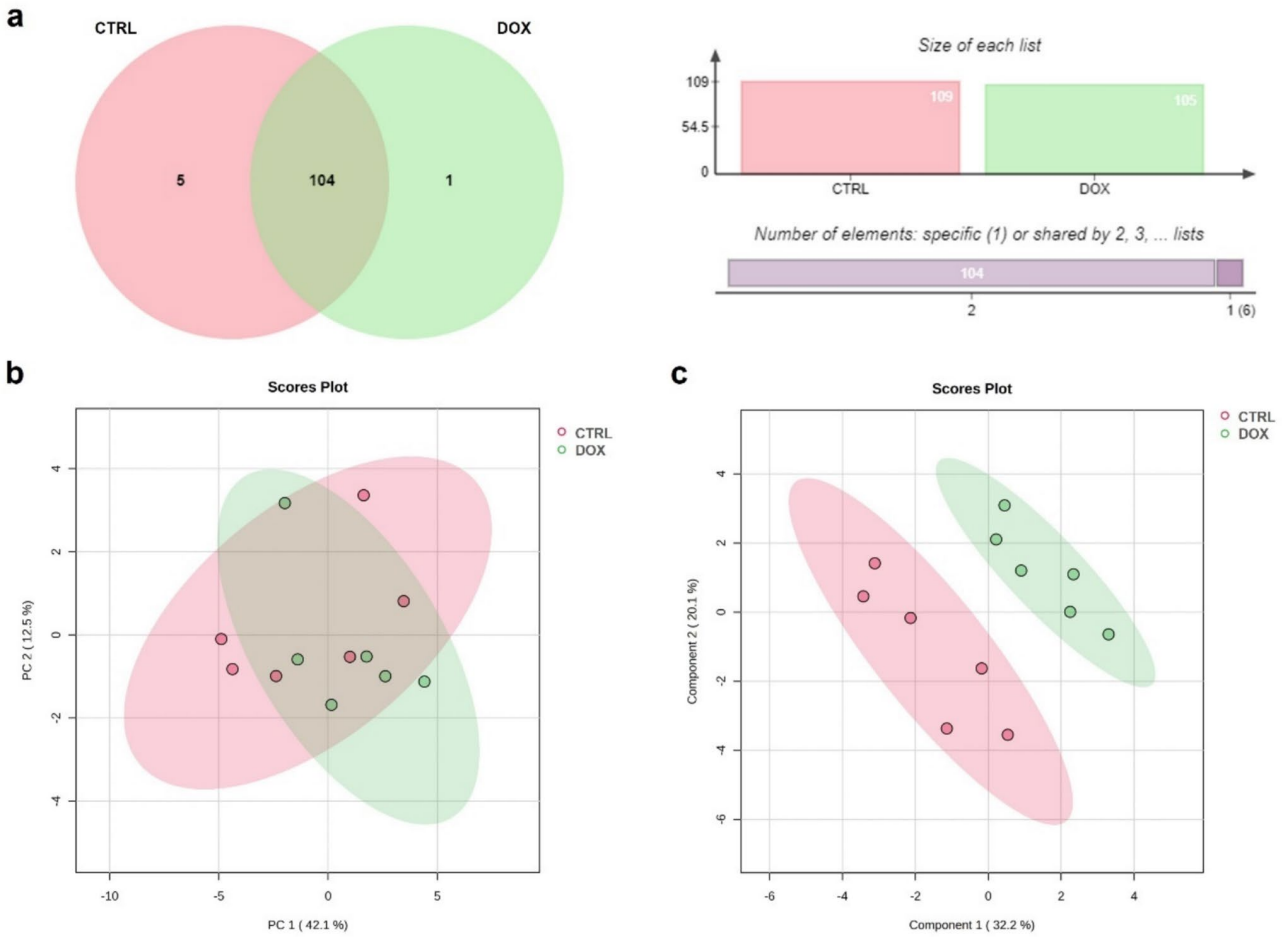
Venn diagram distribution and multivariate analysis of the 110 poly-ubiquitinated proteins identified in at least four out of the six animals considered in each group. Proteins enriched in poly-ubiquitination were obtained by cross-linked glutathione S-transferase (GST)-tandem ubiquitin-binding entities (TUBEs) and then analyzed by SDS-PAGE followed by liquid chromatography coupled to tandem mass spectrometry (GeLC-MS/MS). Venn diagram presenting the proteins most represented in each group (identified in at least four out of the six animals in only one group) and the proteins common between the two groups [**a**]. Proteome dynamics of each group was assessed by principal component analysis (PCA) [**b**] and by partial least squares discriminant analysis (PLS-DA) [**c**]

Considering the impact of DOX in the accumulation of ubiquitinated proteins (Fig. 2), an innovative approach to study cardiac poly-ubiquitinated proteins was implemented, by performing a pull-down of these proteins that were subsequently analyzed by GeLC-MS/MS. This analysis allowed the identification of 318 proteins, in which 110 were identified in at least four animals of each group. The criteria for considering these proteins as poly-ubiquitinated were indicated in the *Materials and Methods* section and a representative spectrum of one modified peptide from a subunit of complex V of oxidative phosphorylation (OXPHOS) is presented in Supplementary Figure S6. The LFQ intensities used for the quantification of these proteins, along with the peptide sequences and protein identification (Uniprot ID, gene and protein names) were included in Supplementary Table S3, while gene name, Uniprot ID, significance/variation, other statistical data, along with the biological process and cellular component, attributed by Gene Ontology of Uniprot database, are indicated for each of the 110 proteins in Supplementary Table S4. The exploration of these proteins in the Venn diagram using the Jvenn tool (Bardou et al. 2014), allowed the identification of five proteins as most represented in the CTRL group, while only one protein was found in the DOX group (Fig. 3a). Moreover, the multivariate analysis of the poly-ubiquitinated proteins (fitting the statistical criteria, e.g. the peptide used for quantification was presented in at least four out of the six animals in each group) showed a slight clustering of groups when applying PCA (Fig. 3b), but a good separation of the two groups for PLS-DA distribution (Fig. 3c).

A closer examination of the poly-ubiquitinated proteins identified as most represented in the CTRL group (Supplementary Table S5) showed that half of them were related to heart sarcomere organization, namely to cardiac muscle contraction (titin) and cytoskeleton organization (LIM domain-binding protein 3). Additionally, heat shock protein beta-1 (HSPB1) acts in the folding of sarcomere proteins, such as titin (Martin and Kirk 2020). These findings advocate for the importance of sarcomeric proteins for the heart structure and function. The two remaining proteins found in the CTRL group were associated with energetic metabolism processes, namely NADH dehydrogenase [ubiquinone] 1 beta subcomplex subunit 4, which is a subunit of complex I of OXPHOS. Similarly, the unique poly-ubiquitinated protein identified as most represented in the DOX group, NADH dehydrogenase [ubiquinone] 1 alpha subcomplex subunit 12 (Supplementary Table S5), belongs to complex I of OXPHOS, which has been associated with increased ROS generation and disrupted redox cycling after DOX treatment (Davies and Doroshow 1986; Renu et al. 2018).

Additionally, the poly-ubiquitinated proteins found with significant differences between DOX and CTRL groups were searched (Table 1). These proteins were annotated using the Volcano plot that is presented in Supplementary Figure S8a. The biological processes associated with these proteins were like those previously mentioned for the proteins detected as most represented in each group. Indeed, two proteins related to heart sarcomere organization, alpha-actinin-2 (ACTN2) and desmin, were found with increased poly-ubiquitination in the DOX group (Table 1). These findings align with the cardiac disorganization and overall heart damage observed after DOX treatment (Fig. 1). Similarly, the glycolytic enzyme glyceraldehyde-3-phosphate dehydrogenase (GAPDH) was also found with higher poly-ubiquitination in DOX (Table 1). Moreover, mitochondrial creatine kinase S-type (Mib-CK), as well as ATP synthase subunit beta (ATPB), cytochrome b-c1 complex subunit 1 (CIII-s1), and succinate dehydrogenase [ubiquinone] flavoprotein subunit (SDHA) belonging to OXPHOS complexes V, III and II, respectively, were found with increased poly-ubiquitination in the DOX group (Table 1). These findings suggest a higher impact of DOX in protein poly-ubiquitination, mainly in mitochondrial proteins, even 2 months after the treatment. Conversely, a decrease in poly-ubiquitination was found in the DOX group for isocitrate dehydrogenase [NAD] subunit alpha (IDHA) that acts in the tricarboxylic acid (TCA) cycle, namely in the conversion of isocitrate to α-ketoglutarate, and trifunctional enzyme subunit beta (TP-B), which is an enzyme involved on fatty acids oxidation (FAO). Both enzymes act in mitochondria, thus corroborating the impact of DOX on mitochondrial proteins.

**Table 1:** Biological processes of the cardiac poly-ubiquitinated proteins found with statistically significant differences (DOX/CTRL comparison)

Biological process	Protein name	Gene name	Uniprot ID
Proteins found with increased poly-ubiquitination in the DOX group*			
Sarcomere organization	Alpha-actinin-2	Actn2	Q9JI91
Sarcomere organization	Desmin	Des	P31001
Glycolysis	Glyceraldehyde-3-phosphate dehydrogenase	Gapdh	P16858
PCr metabolic process	Mitochondrial creatine kinase S-type	Ckmt2	Q6P8J7
OXPHOS	ATP synthase subunit beta	Atp5b	P56480
OXPHOS	Cytochrome b-c1 complex subunit 1	Uqcrc1	Q9CZ13
OXPHOS/TCA cycle	Succinate dehydrogenase [ubiquinone] flavoprotein subunit	Sdha	Q8K2B3
Proteins found with decreased poly-ubiquitination in the DOX group*			
TCA cycle	Isocitrate dehydrogenase [NAD] subunit alpha	Idh3a	Q9D6R2
FAO	Trifunctional enzyme subunit beta	Hadhb	Q99JY0

For each protein detected, the gene name and Uniprot ID are depicted. The proteins were considered when at least four out of the six animals in each group presented the peptide. Experimental groups were statistically compared using an unpaired two-sided *t*-test: *p < 0.05. For further details on statistical data obtained see Supplementary Table S4

*PCr* phosphocreatine, *OXPHOS* oxidative phosphorylation, *TCA* tricarboxylic acid, *FAO* fatty acids oxidation

**Fig. 4:**
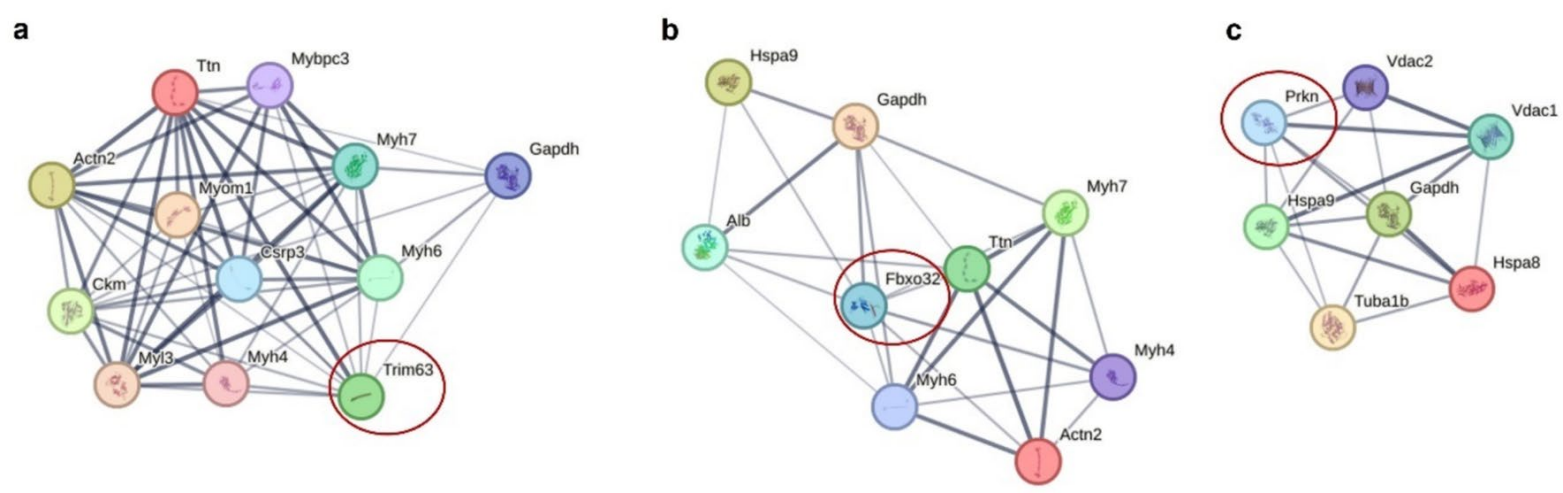
Interactions between the E3 ubiquitin-protein ligases assessed by Western blot (MuRF1, Atrogin-1, Parkin, all indicated by its gene name and marked with a red circle), and the 110 poly-ubiquitinated proteins identified in at least four out of the six animals considered in each group. Proteins enriched in poly-ubiquitination were obtained by cross-linked glutathione S-transferase (GST)-tandem ubiquitin-binding entities (TUBEs) and then analyzed by SDS-PAGE followed by liquid chromatography coupled to tandem mass spectrometry (GeLC-MS/MS). Protein–protein interactions for MuRF1 (Trim63) [**a**], Atrogin-1 (Fbxo32) [**b**], and Parkin (Prkn) [**c**]

As an exploratory and innovative evaluation, the protein–protein interaction (PPI) analysis was performed considering the three E3 ubiquitin-protein ligases assessed by Western blot (MuRF1, Atrogin-1 and Parkin; Fig. 2) and the 110 poly-ubiquitinated proteins identified by GeLC-MS/MS (Supplementary Table S4), using the STRING database (Szklarczyk et al. 2023). The complete PPI contemplating all the proteins is indicated in Supplementary Figure S8b. Figure 4 includes the PPI associated with MuRF1 (Fig. 4a), Atrogin-1 (Fig. 4b), and Parkin (Fig. 4c), identified by the gene names Trim63, Fbxo32, and Prkn, respectively. Curiously, the three E3 ligases share a common protein target, GAPDH, which was found with increased poly-ubiquitination in the DOX group (Table 1). Several interactions were common between MuRF1 and Atrogin-1, including ACTN2, titin, and myosins MYH7, MYH6 and MYH4. Curiously, ACTN2 presented a significant increase in poly-ubiquitination in DOX and titin was among the five poly-ubiquitinated proteins found as most represented in the CTRL group (Supplementary Table S5). MuRF1 is known to interact with proteins related to cardiac muscle contraction (Peris-Moreno et al. 2020), namely myosin-binding protein C (Mybpc3), myomesin-1 (Myom1), cysteine and glycine-rich protein 3 (Csrp3), and myosin light chain 3 (Myl3). Mitochondrial stress-70 protein (HSPA9) was identified as a target of both Atrogin-1 and Parkin. On the other hand, heat shock cognate 71 kDa protein (HSPA8), tubulin alpha-1B chain (Tuba1b), as well as voltage-dependent anion-selective channel proteins 1 (VDAC1) and 2 (VDAC2) were the interactions found for Parkin (Fig. 4c).

**Fig. 5:**
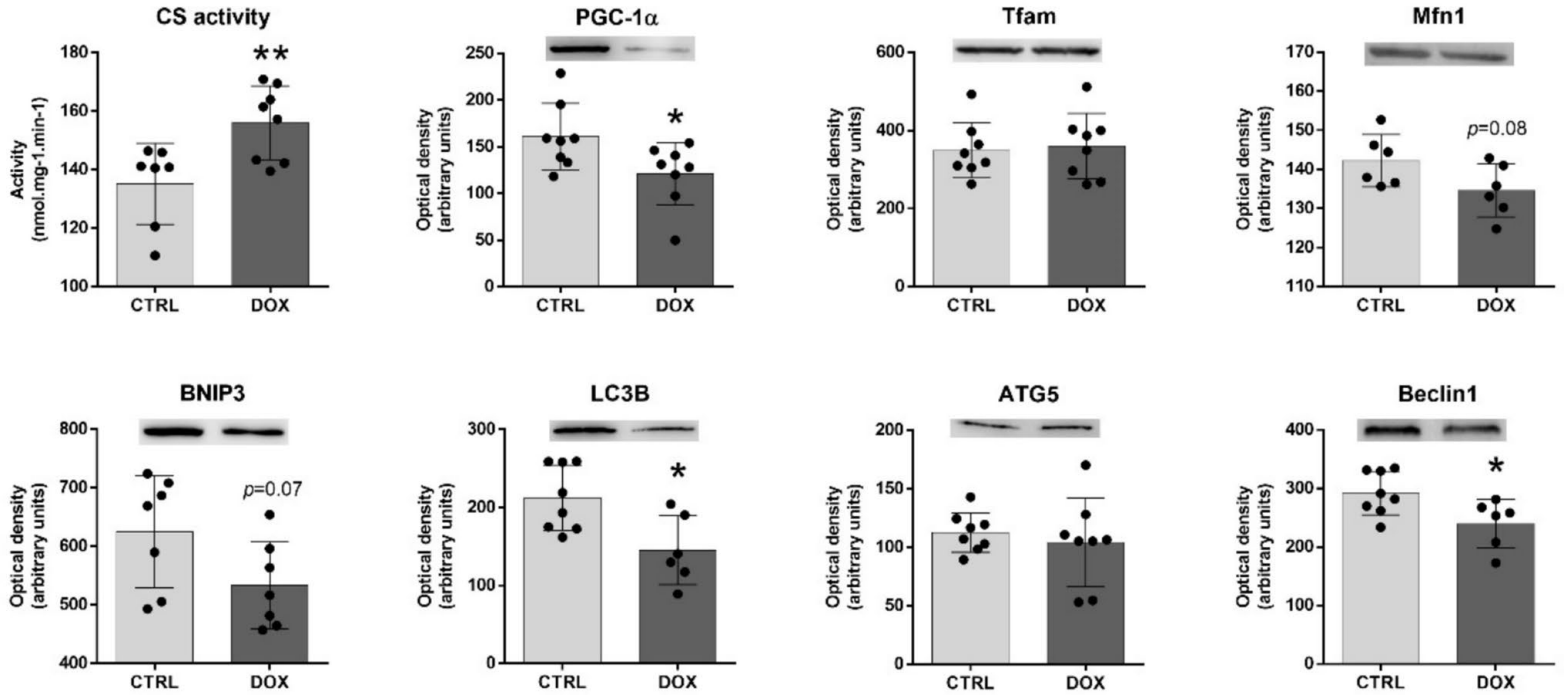
DOX impact on heart markers of mitochondrial dynamics and auto(mito)phagy. The enzymatic activity of CS was determined spectrophotometrically. A representative image of the Western blots obtained is presented. For the total blots obtained, see Supplementary Figure S2. All datapoints are presented overlaying with the mean ± SD (*n* = 6–9) of activity or content. Experimental groups were statistically compared using an unpaired two-sided *t*-test: *p < 0.05, **p < 0.01. *CS* citrate synthase, *PGC-1α* peroxisome proliferator-activated receptor γ coactivator 1 α, *Tfam* mitochondrial transcription factor A, *Mfn1* mitofusin 1, *BNIP3* B-cell lymphoma-2 interacting protein 3, *LC3B* microtubule-associated protein light chain 3, *ATG5* autophagy protein 5

To further explore the mitochondrial modulation induced by DOX, once several proteins related to this organelle were identified as being poly-ubiquitinated, mitochondrial dynamics and auto(mito)phagy markers were assessed (Fig. 5). Indeed, CS activity, which has been considered a rough indicator of mitochondrial density (Larsen et al. 2012), was found increased in the DOX group. Thus, increased mitochondrial density occurred 2 months after the end of DOX treatment. Conversely, the content of PGC-1α was significantly decreased, implying decreased mitochondrial biogenesis, once PGC-1α has an important role in this process, regulating several mitochondrial targets including Tfam and Mfn1 (Kunkel et al. 2016; Ong et al. 2017; Ding et al. 2021). Nonetheless, no differences were observed for Tfam content in the DOX group, indicating no major impact in mitochondrial DNA (mtDNA), and thus, in mitochondrial transcription (Kunkel et al. 2016). On the other hand, a trend towards decreased Mfn1 content was seen (*p* = 0.08), agreeing with the decreased content of PGC-1α. Once Mfn1 acts on mitochondrial fusion (Ong et al. 2017), these findings evoke less fusion of mitochondria and a greater number of this organelle. This outcome is in accordance with increased mitochondrial density, which results from the balance between biogenesis and clearance (Ding et al. 2021). DOX group showed a tendency towards decreased BNIP3 content (*p* = 0.07). This protein acts on the elimination of damaged mitochondria (a process also defined as mitophagy) (Dorn 2010). This finding along with the increased mitochondrial density observed, imply less removal of mitochondria 2 months after DOX administration. In addition, decreased content of autophagy markers LC3B and Beclin1 was found in the DOX group, which may suggest decreased auto(mito)phagy, despite no differences in the ATG5 content, another autophagy marker.

## Discussion

In this study, we investigated the enduring effects of DOX treatment in a non-clinical model featuring elderly animals. The primary emphasis was on the cardiac remodeling associated with protein ubiquitination’ susceptibility and/or accumulation. To the best of our knowledge, this study represents the first attempt to assess the long-term impact of DOX on the UPP and identify the cardiac proteins that are more susceptible to accumulation after poly-ubiquitination. To this end, a mass spectrometry-based proteomics approach was applied to characterize the enriched fraction of poly-ubiquitinated proteins in the aged heart. These novel insights largely enhance the understanding of how the protein ubiquitination status contributes to long-term cardiac remodeling following DOX therapy.

Hearts were collected from aged CD-1 male mice which were chosen to address the translation to the old human population, the most affected by cancer. This population presents a higher incidence of cancer and has a higher predisposition to develop comorbidities that constitute an increased risk for cardiotoxicity, but are misrepresented in cardio-oncology trials (Grann et al. 2006; Hershman et al. 2008; Zamorano et al. 2016; Editorial board 2022; Anker et al. 2024). The cardiac assessments were performed 2 months after the treatment with a clinically relevant cumulative dose (9 mg/kg) of DOX, to evaluate chronic cardiotoxicity. Indeed, four human years have passed since the beginning of the DOX administrations (19 months old in mice equals 75 human years) until the molecular assessments that occurred 2 months after the exposure (22 months old equals 79 years old in humans) (Wang et al. 2020). This long free-drug interval is not common in non-clinical models of cardio-oncology but better reflects the clinical human practice and chronic cardiotoxicity elicited by DOX (Brandão et al. 2021, 2022; Reis-Mendes et al. 2021). Thus, the molecular targets found to be modulated herein imply long-term effects of DOX-induced cardiotoxicity.

Several changes were observed in heart structure (Fig. 1), such as vacuolization of the cytoplasm and the presence of necrotic zones, highlighting cardiac disorganization even 2 months after DOX cessation. These findings demonstrate that even using a low (but pharmacologically relevant) cumulative dose of DOX, cardiac damage persists 2 months after drug exposure, as further explored at the molecular level.

Among the molecular mechanisms contributing to cardiac dysfunction induced by DOX, the accumulation of misfolded proteins, often resulting from irreversible post-translational modifications, has gained more attention over the years (Renu et al. 2018; Montalvo et al. 2020; Brandão et al. 2022; Gaytan et al. 2023). Once these proteins are not properly refolded, they must be cleared out by the proteolytic systems, including the UPP, otherwise they will accumulate (Ghezzi and Bonetto 2003; Butterfield and Dalle-Donne 2014). UPP is the main system for the degradation of contractile proteins, targeting the misfolded proteins and executing their ubiquitination, usually poly-ubiquitination (Zheng and Shabek 2017; Peris-Moreno et al. 2020). Herein, DOX treatment led to higher levels of ubiquitination in proteins (Fig. 2 and Table 1), indicating increased susceptibility and/or accumulation of these proteins. The UPP involves the coordinated action of several enzymes, such as the E3 ubiquitin-protein ligases, from the targeting of misfolded proteins until degradation by the 26S proteasome (Zheng and Shabek 2017; Peris-Moreno et al. 2020). In the present work, a significant decrease in the E3 ubiquitin-protein ligase Atrogin-1 content was observed in the DOX group (Fig. 2), implying a reduction in UPP-mediated proteolysis (Singh et al. 2021). However, no differences were found for the other E3 ubiquitin-protein ligases searched (MuRF1 and Parkin; Fig. 2), which may balance the decreased content of Atrogin-1 and contribute to keep the UPP-mediated proteolysis. Moreover, an overall increase in the ubiquitination levels of proteins was found even 2 months after DOX treatment (Fig. 2). Together, these findings suggest occurrence of ubiquitination in proteins along with decreased proteasome activity, resulting in accumulation of poly-ubiquitinated proteins in cardiac muscle of aged mice 2 months post-DOX administration. Indeed, previous studies have associated the content of Atrogin-1 with the overall proteasomal activity, despite reporting increased outputs (Kumarapeli et al. 2005; Mearini et al. 2008; Yamamoto et al. 2008). However, these studies assessed the UPP components closer to the DOX exposure (6, 24, or 48 h) and used higher doses (15 or 25 mg/kg) compared to our experimental paradigm (Kumarapeli et al. 2005; Yamamoto et al. 2008). Nonetheless, another study, where both in vitro and in vivo models were used, presented decreased proteasome activity accompanied by increased content of proteins associated with proteasome degradation after DOX (Sishi et al. 2013).

The activity of UPP is supported by other proteolytic systems in cardiac muscle, particularly the calpain system. Indeed, calpains are the first proteolytic system to act on myofibrillar proteins, removing them from myofibrils and allowing the subsequent poly-ubiquitination of resulting peptides and their degradation by proteasome (Kachaeva and Shenkman 2012; Martin and Kirk 2020). Interestingly, calpain-3 was among the potential proteases with higher activity in the heart 2 months after DOX treatment (band 1 of zymography; Fig. 2). Calpain-3 targets myofibrillar proteins as titin, the largest protein forming the sarcomere (Kachaeva and Shenkman 2012; Martin and Kirk 2020). Calpain activity was also related to increased proteolysis of desmin, another heart sarcomere protein (Martin and Kirk 2020). Desmin was among the proteins found with increased poly-ubiquitination in the DOX group. This finding together with the increased activity of calpain-3 imply disturbance of the main proteolytic systems of the heart (Montalvo et al. 2020; Gaytan et al. 2023), even 2 months after DOX treatment. Calpain activation was previously associated with DOX-induced cardiac dysfunction in adult female Sprague–Dawley rats, assessed 48 h after being treated with a single i.p. injection of 20 mg/kg of DOX (Min et al. 2015).

Deeper proteomic analysis unveiled that DOX treatment resulted in the accumulation of poly-ubiquitinated mitochondrial proteins (Table 1). This accumulation could be attributed to an elevated misfolding of the proteins specific to this organelle. To gain further insights into the higher susceptibility of mitochondrial proteins to ubiquitination, mitochondrial dynamics was examined by assessing the density of the mitochondrial network (Larsen et al. 2012), as well as markers of biogenesis and auto(mito)phagy (Fig. 5). The data suggest a reduced clearance of mitochondria, even 2 months after DOX therapy. This outcome aligns with the observed accumulation of mitochondrial poly-ubiquitinated proteins. Notably, previous studies have reported accumulation of dysfunctional mitochondria following DOX treatment (Renu et al. 2018; Brandão et al. 2021, 2022). To the best of our knowledge, this study was the first to link the impairment of the UPP system with accumulation of mitochondrial poly-ubiquitinated proteins and disturbed mitochondrial clearance, at least in an aged animal model. This link was further confirmed when analyzing the PPI among MuRF1, Atrogin-1, Parkin, and the poly-ubiquitinated proteins (Fig. 4). The PPI analysis identified specific targets for E3 ubiquitin-protein ligases, thus supporting the different modulation observed for their content. Among the mitochondrial targets, HSPA9 was identified as a substrate of both Atrogin-1 and Parkin. HSPA9, along with other Atrogin-1 targets, were previously associated with increased susceptibility to suffering oxidative modifications after DOX administration (Hung et al. 2020; Brandão et al. 2022). Besides, VDAC1 and VDAC2 were found as substrates of Parkin, which is recognized for its specificity in targeting mitochondrial proteins, primarily those located in the outer membrane (Singh et al. 2021). Nonetheless, most of the targets identified for these E3 ubiquitin-protein ligases are located on myofilaments and cytosol. For instance, all three E3 ligases presented GAPDH as a possible target for ubiquitination. Indeed, this protein was found with increased poly-ubiquitination in DOX, and despite its main function as a glycolytic enzyme, it also acts in sarcomere organization. Furthermore, the majority of common targets among the E3 ubiquitin-protein ligases selected were poly-ubiquitinated proteins related to cardiac muscle contraction and cytoskeleton organization, highlighting their role in heart structure and function (Martin and Kirk 2020; Peris-Moreno et al. 2020).

**Fig. 6:**
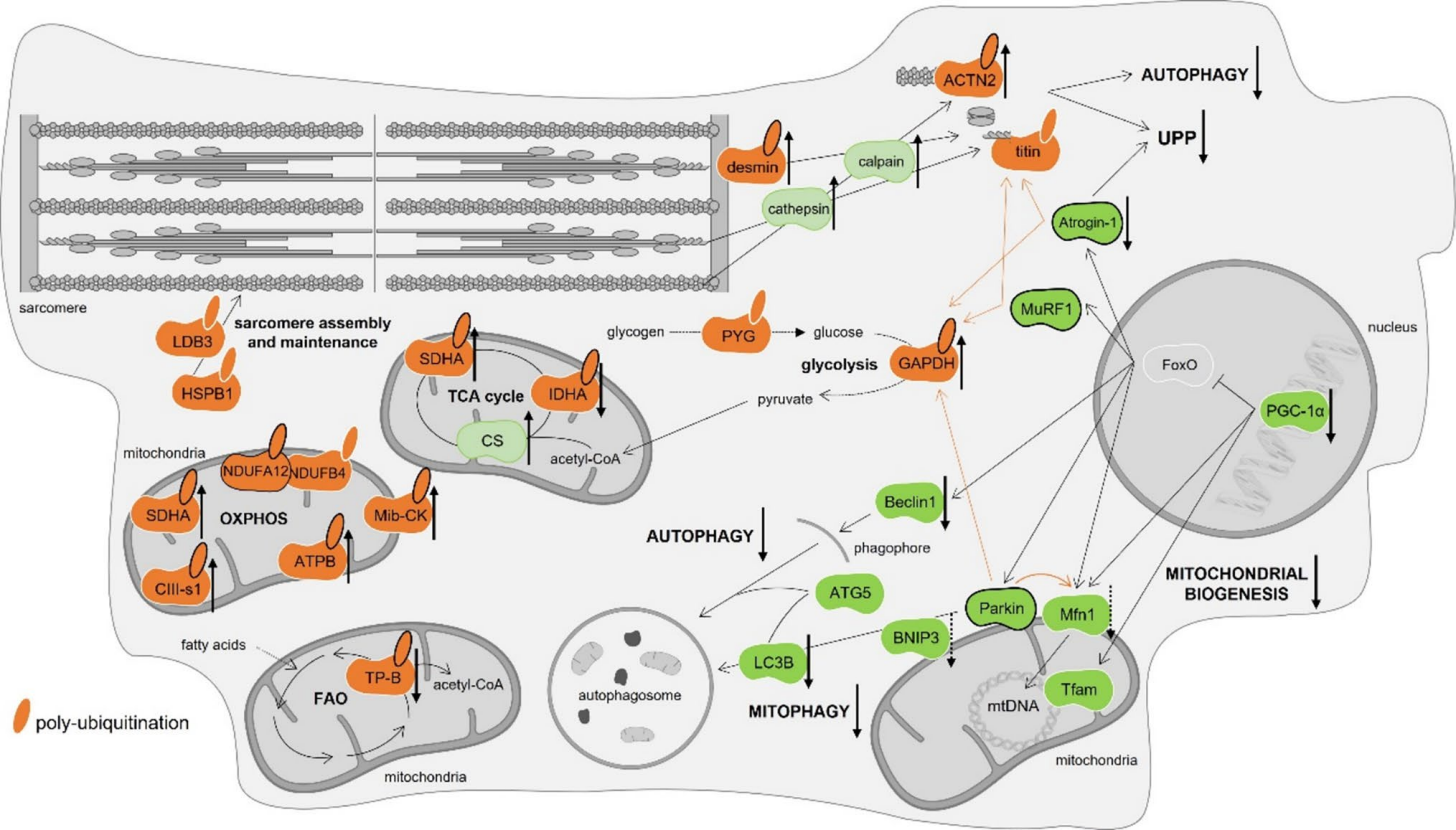
Schematic representation of the overall effect of 9 mg/kg of DOX on cardiac muscle 2 months after the treatment in old CD-1 male mice. Protein content assessed by Western blot is indicated in dark green, with the border of three E3 ubiquitin-protein ligases highlighted in black (Atrogin-1, MuRF1 and Parkin). Proteins’ activity is indicated in light green. Poly-ubiquitinated proteins are indicated in orange with an extra part, with the border of poly-ubiquitinated proteins found as most represented in the CTRL group highlighted in white. The DOX modulation in each protein is denoted with an up arrow (increase effect) or a down arrow (decrease effect). Figure made with *Servier medical art*. *ACTN2* alpha-actinin-2, *ATG5* autophagy protein 5, *ATPB* ATP synthase subunit beta, *BNIP3* B-cell lymphoma-2 interacting protein 3, *CIII-s1* cytochrome b-c1 complex subunit 1, *CS* citrate synthase, *FAO* fatty acids oxidation, *GAPDH* glyceraldehyde-3-phosphate dehydrogenase, *HSPB1* heat shock protein beta-1, *IDHA* isocitrate dehydrogenase [NAD] subunit alpha, *LC3B* microtubule-associated protein light chain 3, *LDB3* LIM domain-binding protein 3, *Mfn1* mitofusin1, *Mib-CK* mitochondrial creatine kinase S-type, *mtDNA* mitochondrial DNA, *MuRF1* muscle RING finger protein 1, *NDUFA12* NADH dehydrogenase [ubiquinone] 1 alpha subcomplex subunit 12, *NDUFB4* NADH dehydrogenase [ubiquinone] 1 beta subcomplex subunit 4, *OXPHOS* oxidative phosphorylation, *FoxO* forkhead box O, *PGC-1α* peroxisome proliferator-activated receptor γ coactivator 1 α, *PYG* glycogen phosphorylase, *SDHA* succinate dehydrogenase [ubiquinone] flavoprotein subunit, *TCA* tricarboxylic acid, *Tfam* mitochondrial transcription factor A, *TP-B* trifunctional enzyme subunit beta, *UPP* ubiquitin–proteasome pathway

To conclude, DOX treatment not only affected the cardiac structure but also influenced the ubiquitination status of cardiac proteins even 2 months after its administration. The increased activity of proteases, such as calpains and cathepsins, was associated with the accumulation of poly-ubiquitinated proteins in the aged heart following DOX exposure. Most of these poly-ubiquitinated proteins were primarily related to the heart sarcomere organization and pathways associated with energetic mitochondrial metabolism. The impact of DOX on mitochondrial dynamics was further supported by the increased number of this organelle, emphasizing accumulation of dysfunctional mitochondria, likely due to decreased auto(mito)phagy despite reduced biogenesis, as illustrated in Fig. 6.

## Supplementary Information

Below is the link to the electronic supplementary material.

Supplementary file1 (PDF 2021 KB)

Supplementary file2 (XLSX 142 KB)

## Data Availability

The mass spectrometry proteomics datasets generated and/or analyzed during the current study are available in the ProteomeXchange Consortium via the PRIDE partner repository with the dataset identifier PXD048078. Other datasets generated and/or analyzed during the current study are available from the corresponding author on reasonable request.
