# Supplementary material for: Comprehensive ubiquitome analysis reveals persistent mitochondrial remodeling disruptions from doxorubicin-induced cardiotoxicity in aged CD-1 male mice
Source: Arch Toxicol. 2025 Mar 4;99(6):2447–62. doi: 10.1007/s00204-025-04006-2 (PMC12185556; doi:10.1007/s00204-025-04006-2)

Comprehensive ubiquitome analysis reveals persistent mitochondrial remodeling disruptions from doxorubicin-induced cardiotoxicity in aged CD-1 male mice

Archives of Toxicology

Sofia Reis Brandão[1,2,3,*], Elisa Lazzari, Rui Vitorino, Germana Meroni, Ana Reis-Mendes, Maria João Neuparth, Francisco Amado, Félix Carvalho, Rita Ferreira, Vera Marisa Costa[1,2,*]

* Corresponding authors:

Sofia Reis Brandão, ORCID ID: https://orcid.org/0000-0001-9964-8947; E-mail address: sofiarbrandao@ua.pt,

Vera Marisa Costa, ORCID ID: https://orcid.org/0000-0002-0471-2756; E-mail address: veramcosta@ff.up.pt,

Postal address: UCIBIO/REQUIMTE, Laboratório de Toxicologia, Faculdade de Farmácia, Universidade do Porto, Rua de Jorge Viterbo Ferreira, 228, 4050-313, Porto, Portugal.

[1]Associate Laboratory i4HB - Institute for Health and Bioeconomy, Faculty of Pharmacy, University of Porto, 4050-313 Porto, Portugal;
[2]UCIBIO-Applied Molecular Biosciences Unit, Laboratory of Toxicology, Department of Biological Sciences, Faculty of Pharmacy, University of Porto, 4050-313 Porto, Portugal;
[3]LAQV-REQUIMTE, Department of Chemistry, University of Aveiro, 3810-193 Aveiro, Portugal.

Supplementary Figure S1. Ponceau S images obtained in cardiac homogenates for ATG5, Atrogin-1, Beclin1, BNIP3, C/EBPβ, CITED4, LC3B, Mfn1, MuRF1, Parkin, PGC-1α, SCFR, and Tfam, along with protein ubiquitination levels assessed by Western blot. ATG5: autophagy protein 5, BNIP3: B-cell lymphoma-2 interacting protein 3, C/EBPβ: CCAAT/enhancer-binding protein β, CITED4: Cbp/p300-interacting transactivator 4, LC3B: microtubule-associated protein light chain 3, Mfn1: mitofusin 1, MuRF1: muscle RING finger protein 1, PGC-1α: peroxisome proliferator-activated receptor γ coactivator 1 α, SCFR: mast/stem cell growth factor receptor Kit, Tfam: mitochondrial transcription factor A.

Supplementary Figure S2. Western blot images obtained in cardiac homogenates for ATG5, Atrogin-1, Beclin1, BNIP3, C/EBPβ, CITED4, LC3B, Mfn1, MuRF1, Parkin, PGC-1α, SCFR, and Tfam, along with protein ubiquitination and carbonylation levels. ATG5: autophagy protein 5, BNIP3: B-cell lymphoma-2 interacting protein 3, C/EBPβ: CCAAT/enhancer-binding protein β, CITED4: Cbp/p300-interacting transactivator 4, LC3B: microtubule-associated protein light chain 3, Mfn1: mitofusin 1, MuRF1: muscle RING finger protein 1, PGC-1α: peroxisome proliferator-activated receptor γ coactivator 1 α, SCFR: mast/stem cell growth factor receptor Kit, Tfam: mitochondrial transcription factor A.

Supplementary Figure S3. 10% SDS-PAGE gel images were obtained to determine the proteolytic activity in cardiac homogenates. The active proteolytic bands are indicated [**a**] as well as the gels incubated with inhibitors of MMPs indicating no major contribution of these proteins to the proteolytic activity [**b**]. The molecular weight, in kDa, of each band of the molecular weight marker (MWM) observed is depicted.

Supplementary Figure S4. Western blot images were obtained for the several fractions (IP, FT, G-T, and G) collected during the pull-down of poly-ubiquitinated proteins using GST-TUBEs for each sample. The images were obtained after incubation with ubiquitin antibody. MWM: molecular weight marker, IP: input of cardiac homogenates, FT: unbound fraction of GST-TUBEs, G-T: GST-TUBEs, G: GST alone used as the methodological negative control.

Supplementary Figure S5. 12.5% SDS-PAGE gel images obtained after separation of poly-ubiquitinated proteins enriched by applying cross-linked GST-TUBEs protocol in cardiac homogenates. Considering the four fractions for each sample during the protocol, only GST-TUBEs (G-T of Supplementary Figure S4) were used (n = 6). MWM: molecular weight marker, SB: sample buffer.

Supplementary Figure S6. Spectra manual inspection of a poly-ubiquitinated peptide from ATP synthase alpha chain (gene name: Atp5a1, Uniprot ID: Q03265). MS/MS spectrum highlighting the modified Lys amino acid residue (GlyGly), identified as **Kg** [**a**]. The amino acid residue position in the 3D protein structure (retrieved from Alphafold) is also indicated [**b**].

Supplementary Figure S7. Effect of DOX treatment on cardiac markers related to heart regeneration (SCFR, C/EBPβ and CITED4) measured by Western blot [**a**] and on each band detected for cardiac protein ubiquitination and carbonylation levels along with proteolytic activity measured by zymography [**b**]. For each protein, a representative image of the blot obtained is presented. For the total blots and proteolytic gels obtained check Supplementary Figure S2 and S3, respectively. All datapoints are presented overlaying with the mean ± SD of content (n = 6-8). The groups were compared using an unpaired two-sided t-test: *$p<0.05$, **$p<0.01$. SCFR: mast/stem cell growth factor receptor Kit, C/EBPβ: CCAAT/enhancer-binding protein β, CITED4: Cbp/p300-interacting transactivator 4.

Supplementary Figure S8. Effect of DOX treatment on cardiac poly-ubiquitinated proteins. Volcano plot [**a**] was used for the identification of poly-ubiquitinated proteins with statistically significant differences (identified in at least four out of the six animals in the two groups) between DOX and CTRL (*$p<0.05$ DOX vs CTRL). Protein-protein interaction [**b**] analysis between the E3 ubiquitin-protein ligases assessed by Western blot (MuRF1, Atrogin-1, Parkin; identified by its gene name and marked with a red circle) and the 110 poly-ubiquitinated proteins identified in at least four out of the six animals considered in each group. Proteins enriched in poly-ubiquitination were obtained by cross-linked glutathione S-transferase (GST)-tandem ubiquitin-binding entities (TUBEs) and then analyzed by SDS-PGAE followed by liquid chromatography coupled to tandem mass spectrometry (GeLC-MS/MS). The proteins are indicated by their gene name.

Supplementary Figure S1

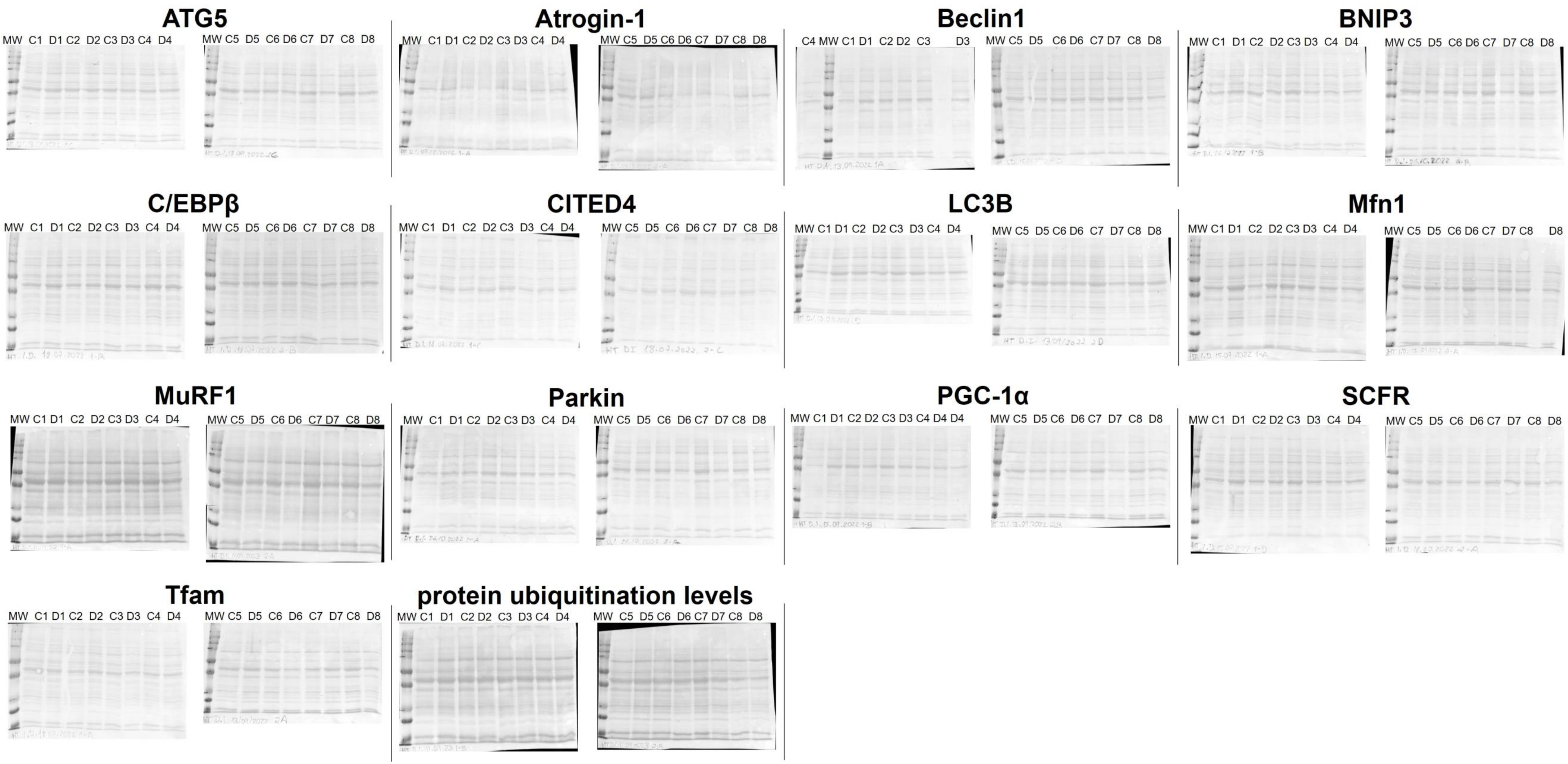

Supplementary Figure S2

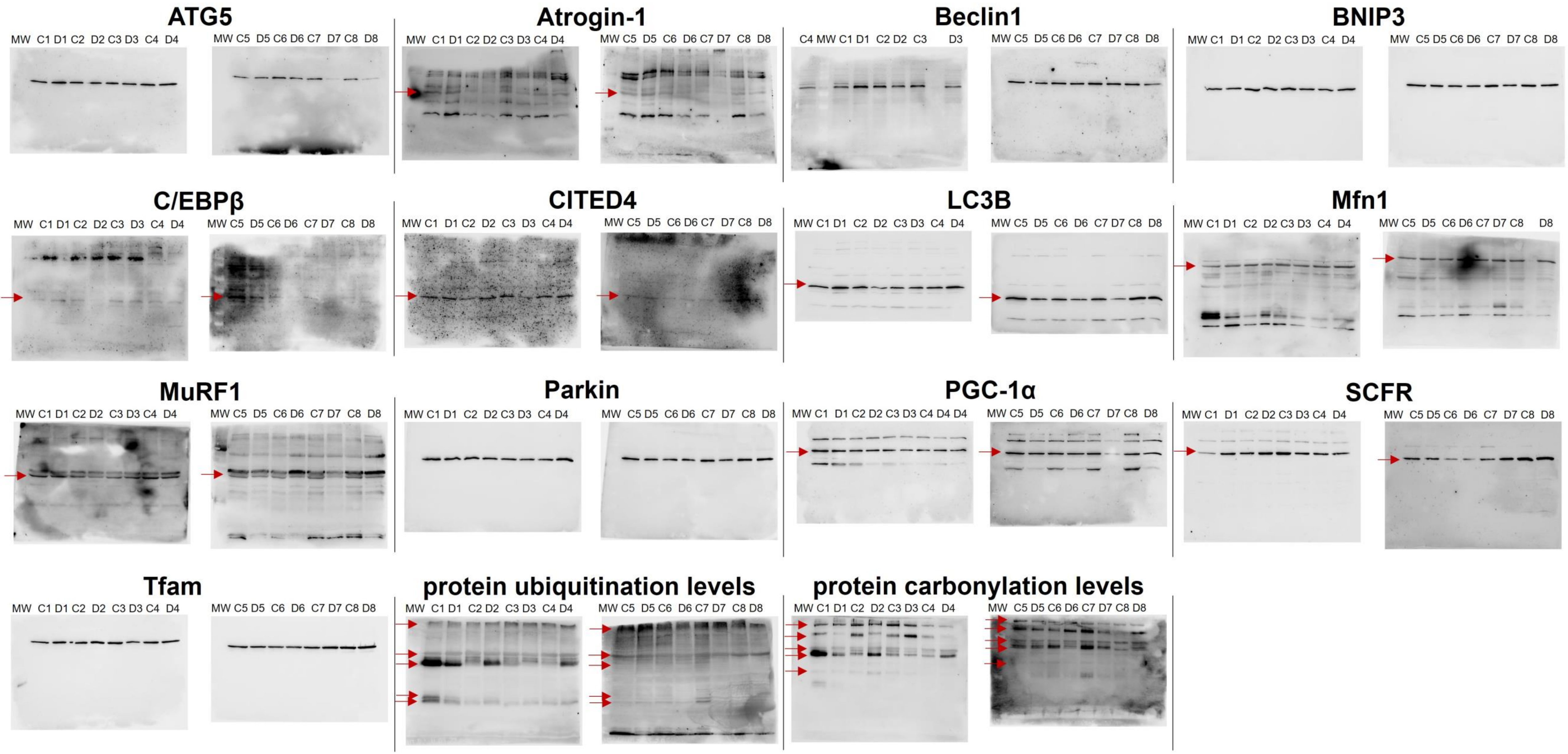

Supplementary Figure S3

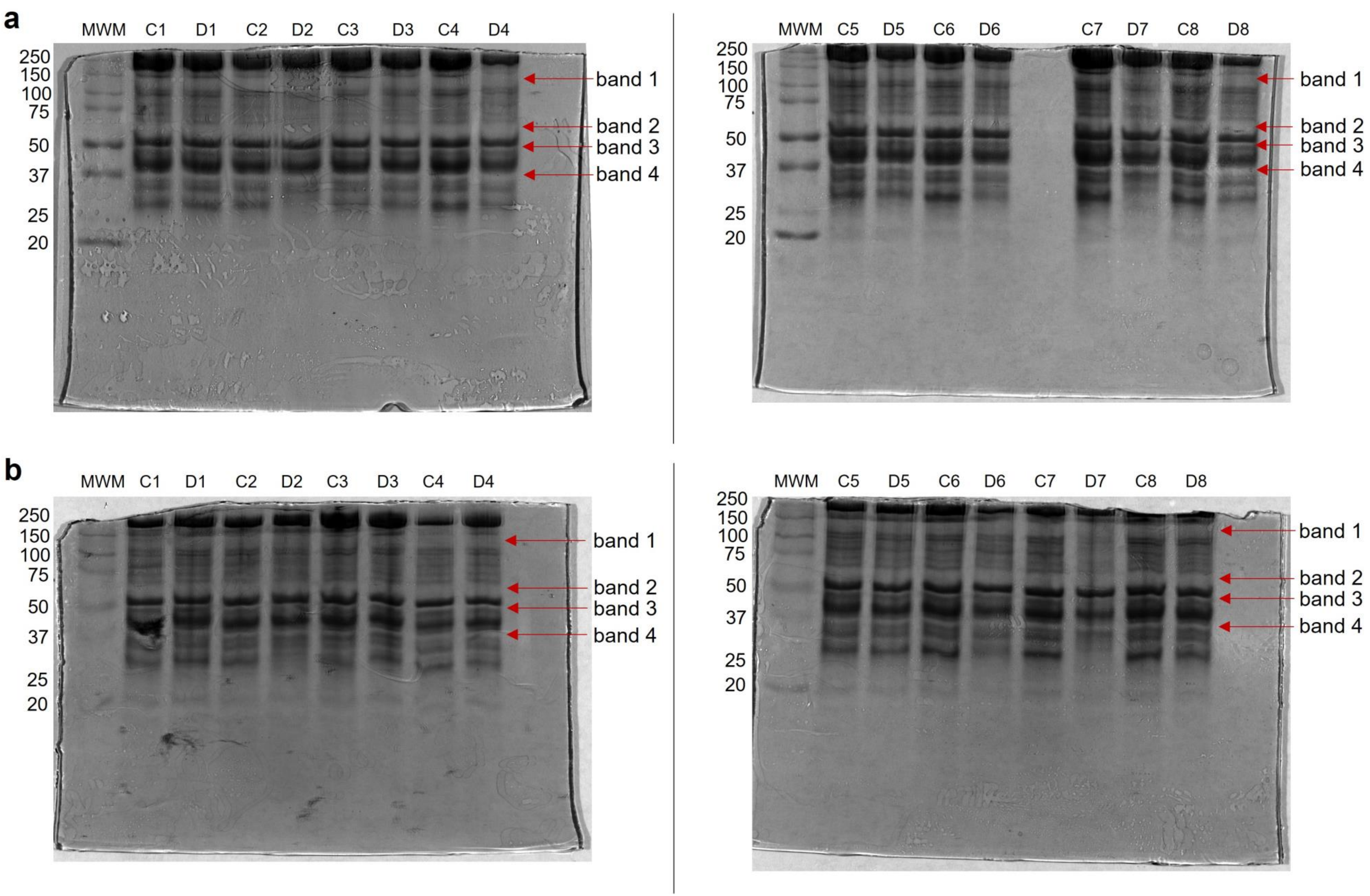

Supplementary Figure S4

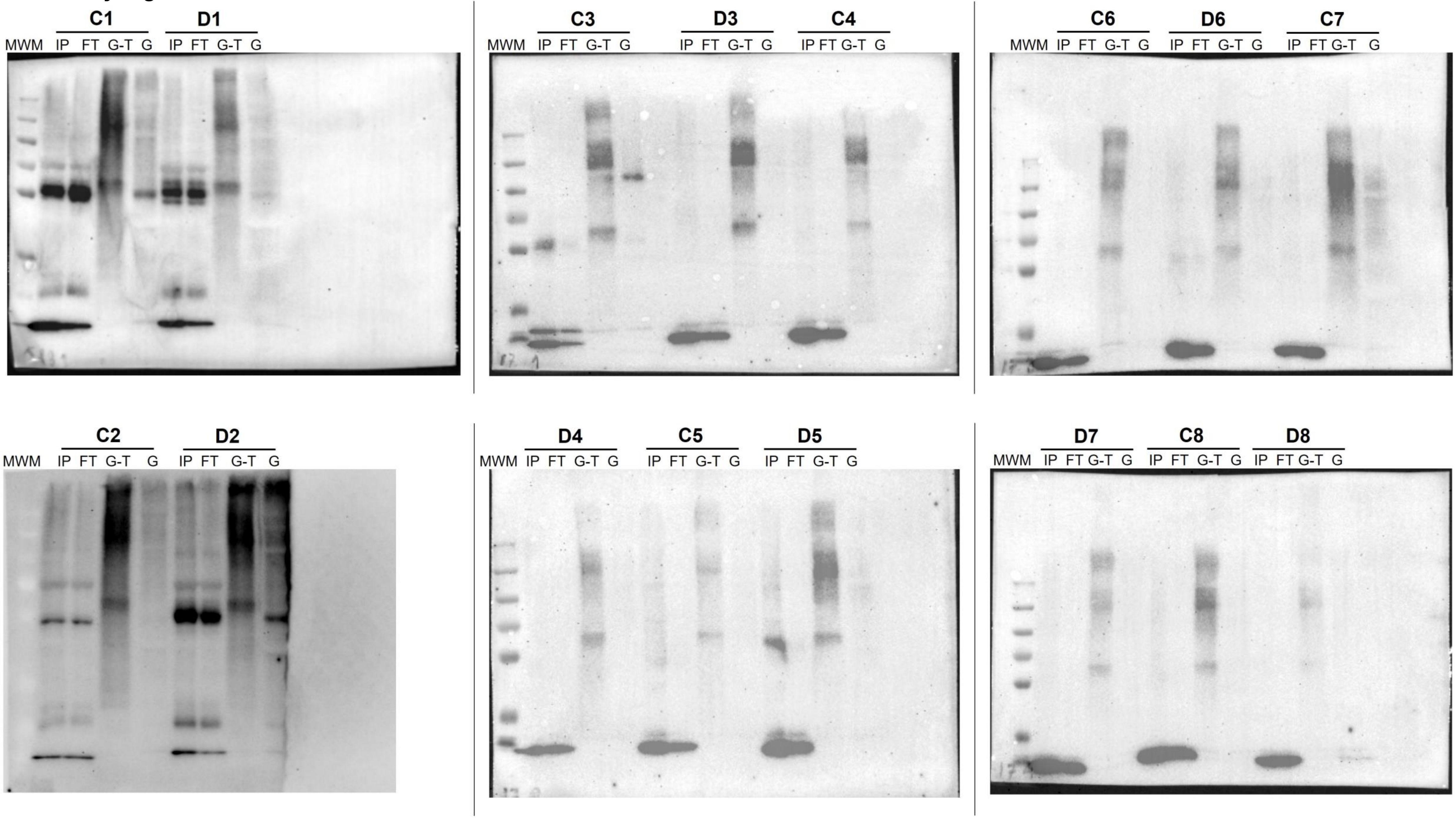

Supplementary Figure S5

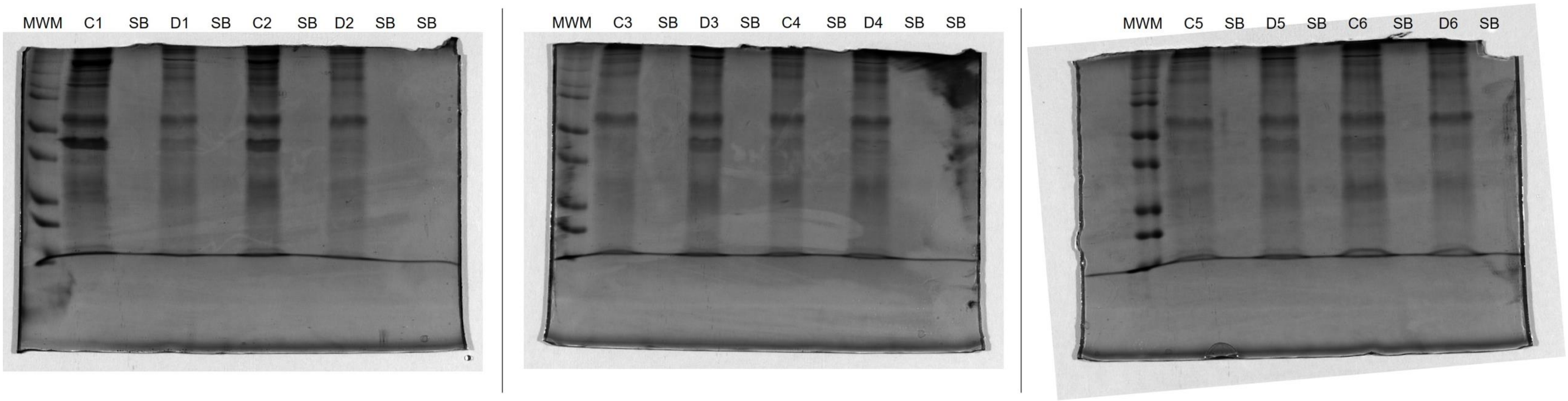

Supplementary Figure S6

a

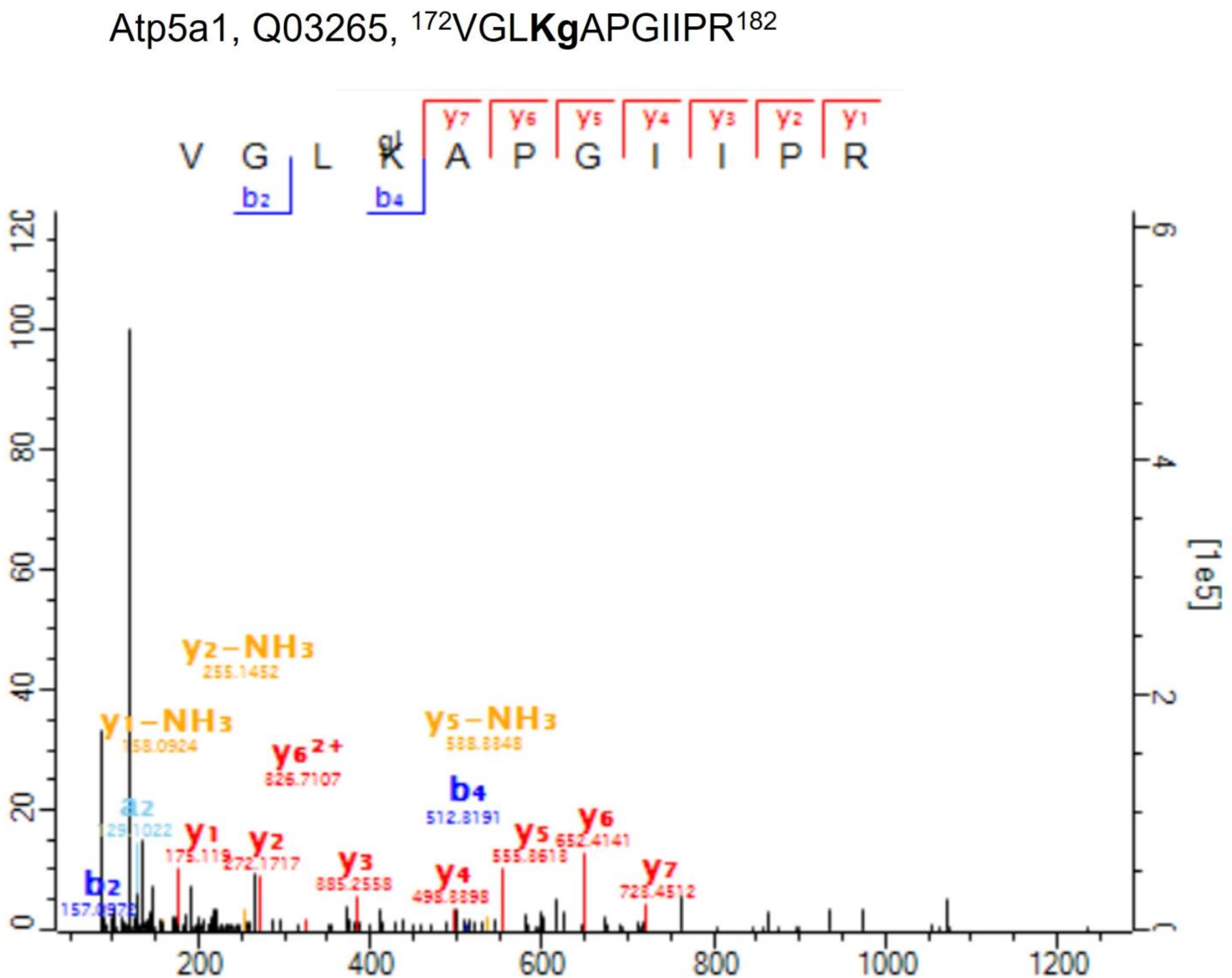

b

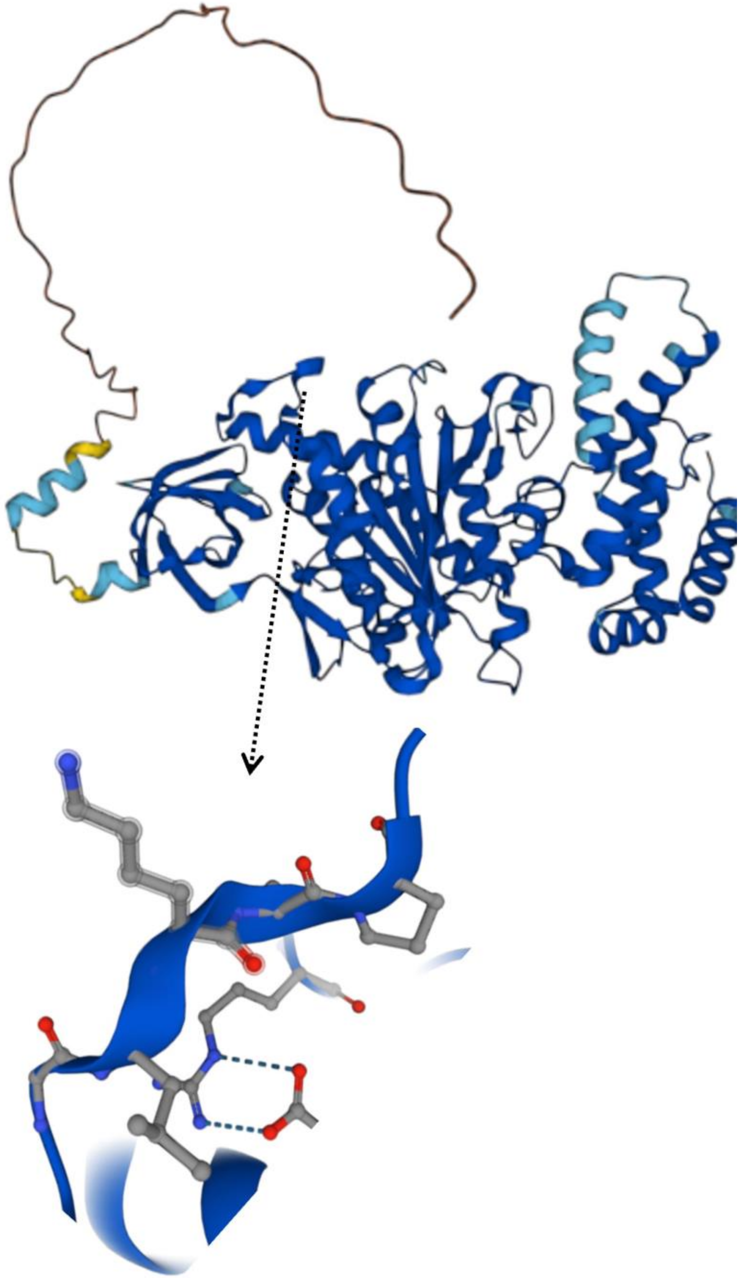

Supplementary Figure S7

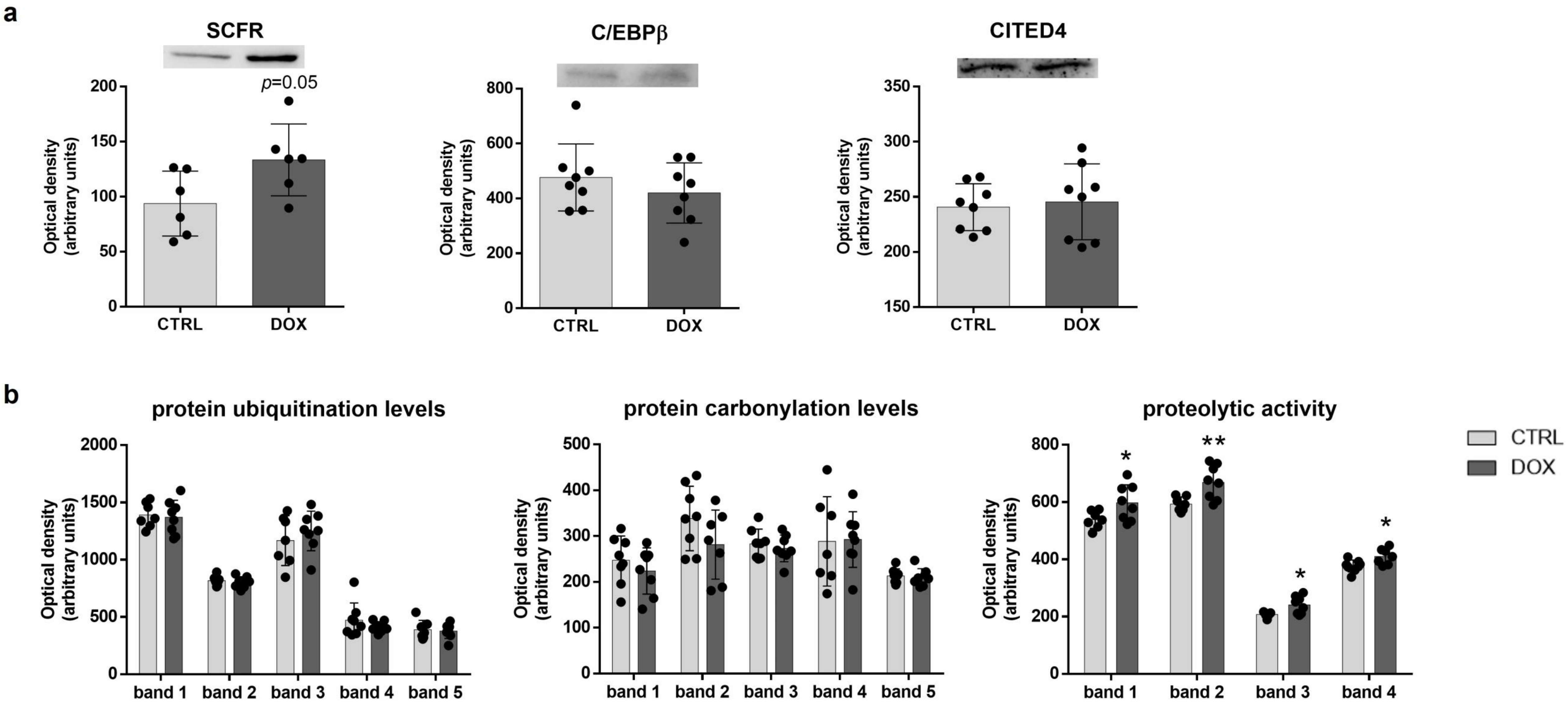

Supplementary Figure S8

a

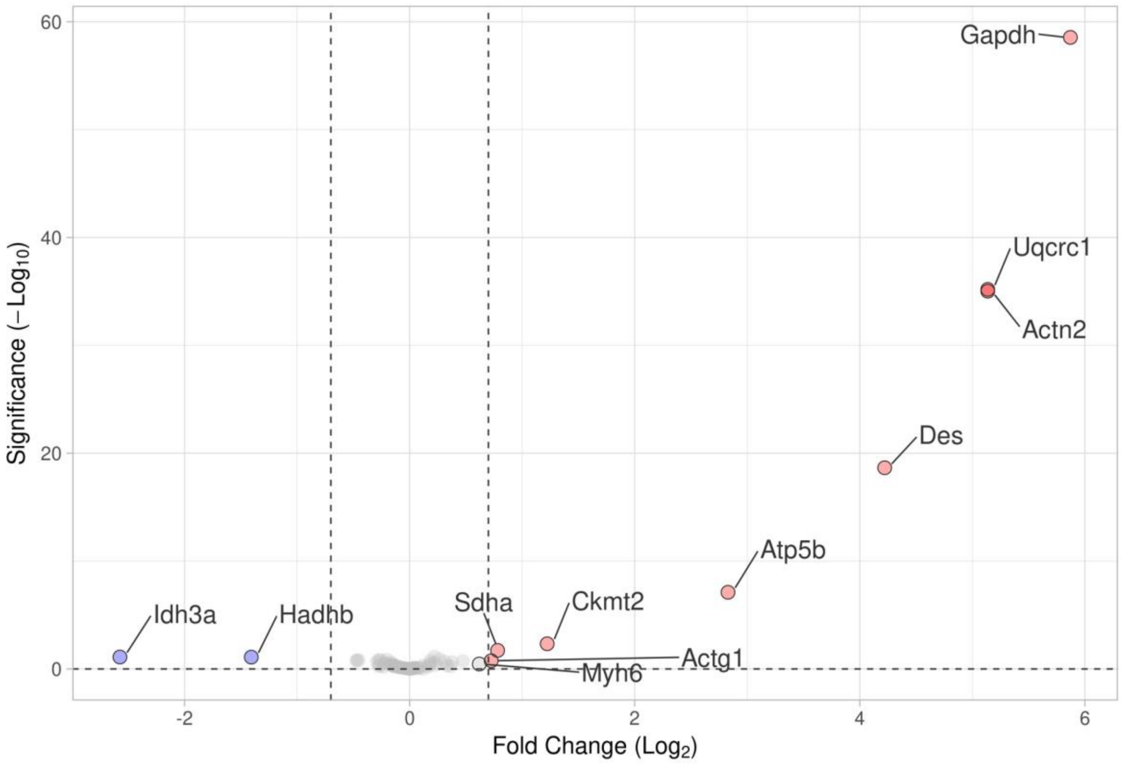

b

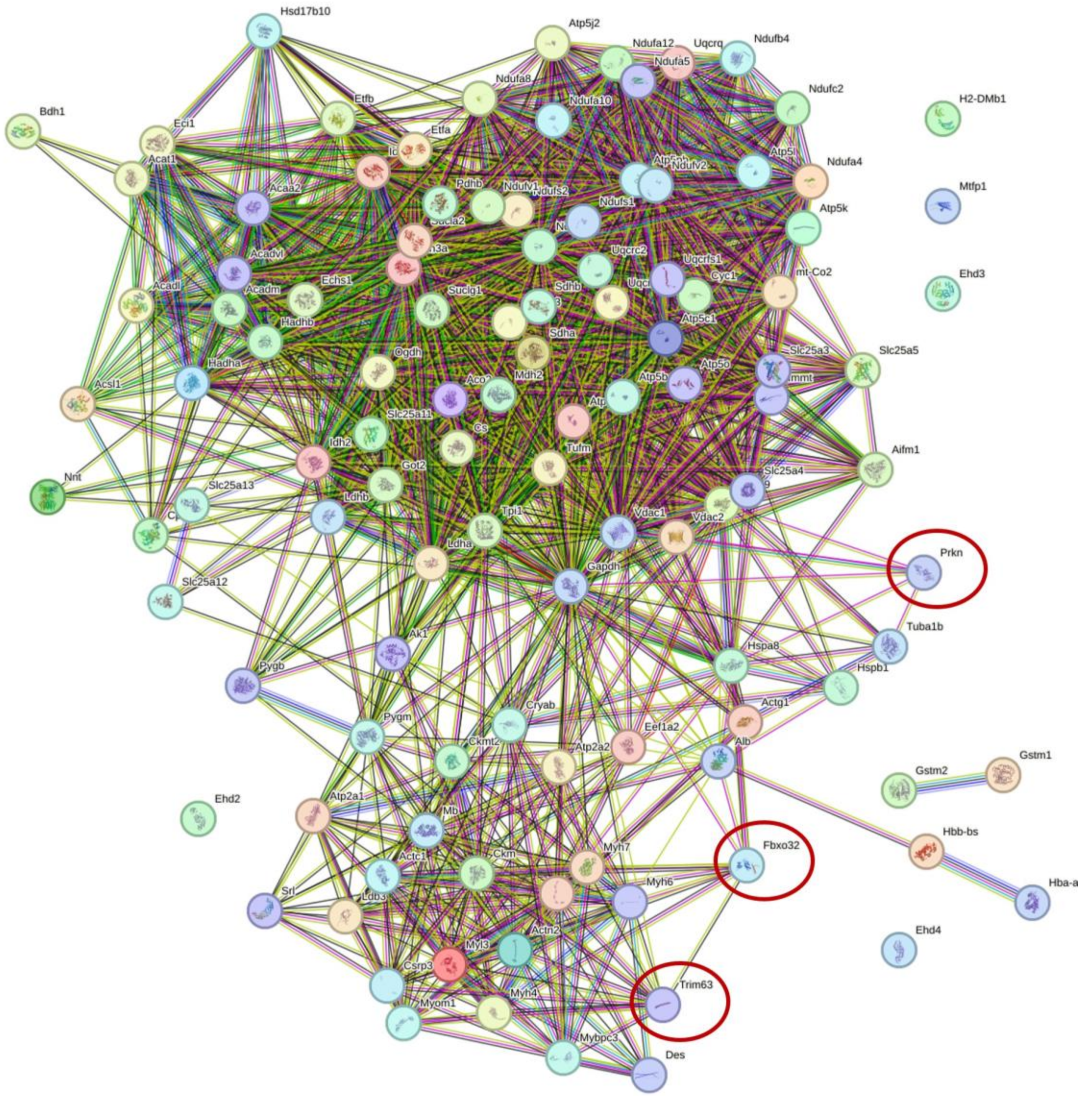

Supplement: Supplementary file 1 — Supplementary file1 (PDF 2021 KB) [file 204_2025_4006_MOESM1_ESM.pdf]
